# Targeted neutrophil-mimetic liposomes promote cardiac repair by adsorbing proinflammatory cytokines and regulating the immune microenvironment

**DOI:** 10.1186/s12951-022-01433-6

**Published:** 2022-05-07

**Authors:** Jing Chen, Yanan Song, Qiaozi Wang, Qiyu Li, Haipeng Tan, Jinfeng Gao, Ning Zhang, Xueyi Weng, Dili Sun, Wusiman Yakufu, Zhengmin Wang, Juying Qian, Zhiqing Pang, Zheyong Huang, Junbo Ge

**Affiliations:** 1 Department of Cardiology, Zhongshan Hospital, Shanghai Institute of Cardiovascular Diseases, Fudan University, 180 Fenglin Road, Shanghai, 200032 China; 2 School of Pharmacy, Key Laboratory of Smart Drug Delivery, Ministry of Education, Fudan University, 826 Zhangheng Road, Shanghai, 201203 China

**Keywords:** Neutrophil biomimetic, Targeted delivery, Inflammation neutralization, Myocardial ischemia–reperfusion injury

## Abstract

Acute myocardial infarction (MI) induces a sterile inflammatory response that may result in poor cardiac remodeling and dysfunction. Despite the progress in anti-cytokine biologics, anti-inflammation therapy of MI remains unsatisfactory, due largely to the lack of targeting and the complexity of cytokine interactions. Based on the nature of inflammatory chemotaxis and the cytokine-binding properties of neutrophils, we fabricated biomimetic nanoparticles for targeted and broad-spectrum anti-inflammation therapy of MI. By fusing neutrophil membranes with conventional liposomes, we fabricated biomimetic liposomes (Neu-LPs) that inherited the surface antigens of the source cells, making them ideal decoys of neutrophil-targeted biological molecules. Based on their abundant chemokine and cytokine membrane receptors, Neu-LPs targeted infarcted hearts, neutralized proinflammatory cytokines, and thus suppressed intense inflammation and regulated the immune microenvironment. Consequently, Neu-LPs showed significant therapeutic efficacy by providing cardiac protection and promoting angiogenesis in a mouse model of myocardial ischemia–reperfusion. Therefore, Neu-LPs have high clinical translation potential and could be developed as an anti-inflammatory agent to remove broad-spectrum inflammatory cytokines during MI and other neutrophil-involved diseases.

**Graphical Abstract:**

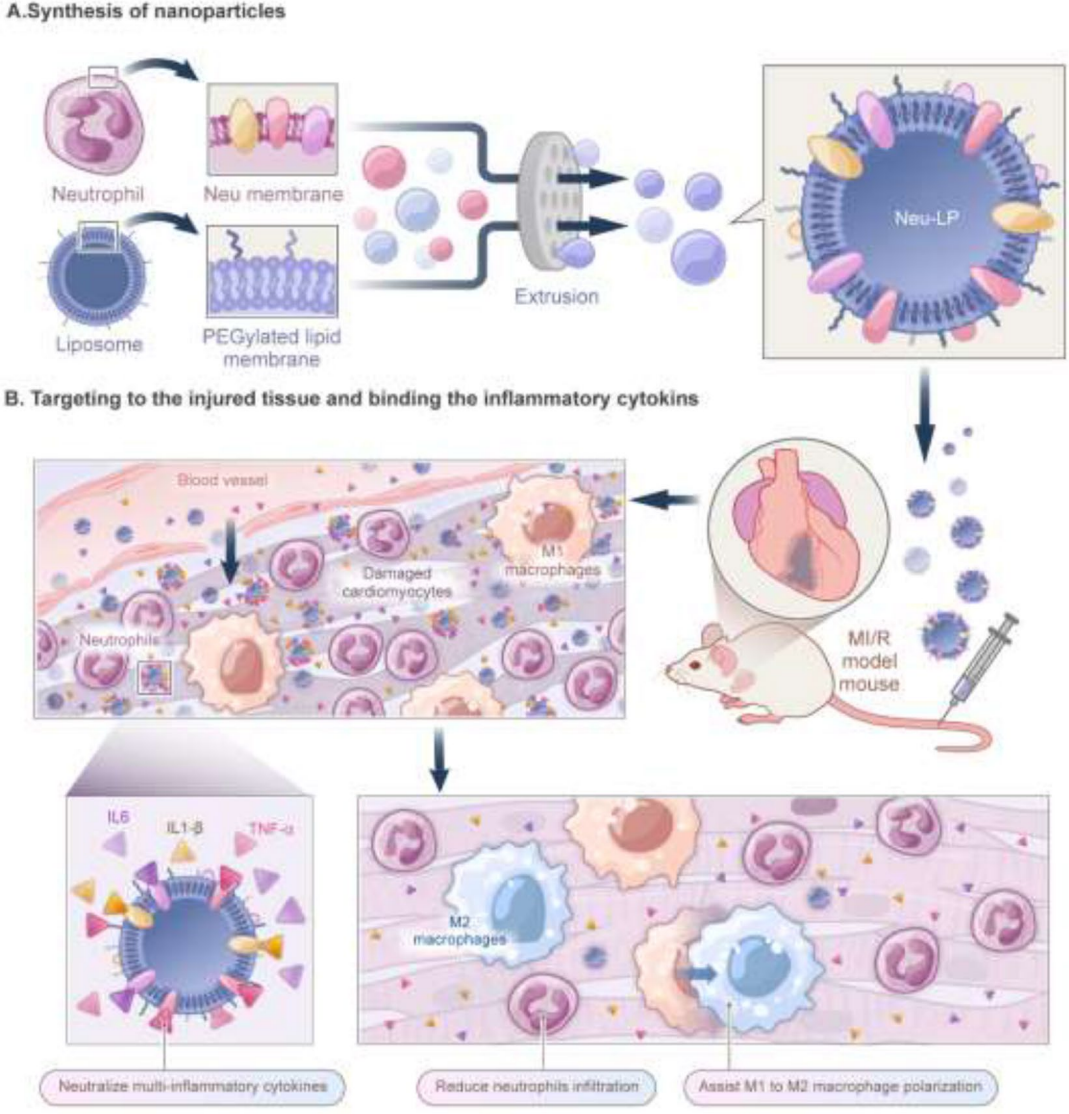

**Supplementary Information:**

The online version contains supplementary material available at 10.1186/s12951-022-01433-6.

## Introduction

Myocardial infarction (MI) is a major cause of morbidity and mortality worldwide [1, 2]. Though reperfusion therapy has significantly reduced the acute mortality of MI, patients who survive the acute event remain at risk of developing heart failure. At the onset of MI, an intense inflammatory response is triggered that clears dead cells and matrix debris from infarcted hearts [3–5]. Though early inflammatory activation is a necessary event for cardiac repair, excessive, prolonged, and dysregulated inflammation has been implicated in the pathogenesis of complications and may be involved in the development of heart failure following infarction [6–9], making it a potential therapeutic target for improving clinical outcomes in patients with MI.

A variety of proinflammatory cytokines, such as tumor necrosis factor alpha (TNFα), interleukin 1 beta (IL1β), and interleukin 6 (IL6), are upregulated and secreted early after MI and play an important role in the post-infarction inflammatory response. These cytokines may produce cytotoxic effects and further contribute to myocardial injury, suggesting that they are potential targets for the development of anti-inflammation therapies [10–12]. However, despite promising results shown in animal models, clinical studies of therapies targeting inflammatory cytokines as a strategy for cardioprotection have produced mixed results. The reasons for the many failures are unclear but may be related to the limitations of using a single-target approach directed to the proinflammatory proponent of acute MI. Pathological inflammation after MI is orchestrated by a large number of molecules, and inhibition of one or a few may not be sufficient to halt or reverse disease progression owing to the multiplicity of cytokine targets and the complexity of cytokine interactions. Additionally, the toxicity of cytokine inhibitors remains highly unpredictable and may increase the risk of fatal infections due to their lack of targeting capacity, which blunts the body’s local and systemic inflammatory response to infection. Therefore, alternative targeted and broad-spectrum approaches that enhance drug accumulation in the site of disease and overcome the complexity and heterogeneity of the inflammatory network are highly desirable for the treatment of inflammation after MI.

Recently, cell-mimetic nanoparticles have been explored as a promising strategy for detoxing [13] and neutralizing inflammatory cytokines [14, 15]. Because of their natural cell membrane coatings, these nanoparticles possess a similar surface protein profile as the source cells and could neutralize inflammatory cytokines that rely on abundant cytokine receptors on the cell surface [16]. The advancement of biomimetic nanoparticles for the neutralization of broad-spectrum cytokines further promises an anti-inflammatory strategy to address the challenges of cardiac repair [17].

As the first immune cell subset to respond to cardiac injury, neutrophils play an important role in the acute inflammatory response after MI [18, 19]. Neutrophils are immediately activated and recruited into infarcted myocardium, peaking at 3 days and continuing to accumulate over 7–14 days after MI onset [20]. Once recruited to the ischemic area, their rapid degradation and degranulation propagates the acute inflammatory response to neighboring areas of the myocardium (so-called ‘neutrophil-induced injury’) and triggers infiltration of inflammatory cells into the ischemic tissue, representing an important step in the local amplification of the initial inflammatory response [11]. Various proinflammatory cytokines and chemokines have been identified in damaged myocardium, which play important roles in neutrophil migration and self-amplification [21, 22]. Moreover, treatment strategies that reduce the accumulation or inhibit the activity of neutrophils have been shown to reduce early and late myocardial infarct size following MI [23].

Inspired by these findings, we developed a neutrophil-mimetic liposome (Neu-LP) as a broad-spectrum anti-inflammatory agent for MI. By fusing liposomes (LPs) with neutrophil membrane vesicles (NMVs), we generated Neu-LPs that inherited abundant cytokine and chemokine receptors from the source cells. We hypothesized that, by taking advantage of the infarct-homing ability of neutrophils, Neu-LPs would transport into the damaged myocardium and concurrently neutralize inflammatory cytokines, modulate inflammatory responses in the cardiac microenvironment, and thus improve MI healing. In this study, we examined the targeting ability of Neu-LPs in vitro and in vivo, and we verified their broad-spectrum cytokine neutralization capacity and inflammation modulation function by measuring the cytokine profile and surface marker expressions. The effects of Neu-LP injection on cardiac protection and angiogenesis were examined in a mouse model of acute myocardial ischemia–reperfusion (MI/R).

## Results and discussion

### Preparation and characterization of Neu-LPs

**Fig. 1:**
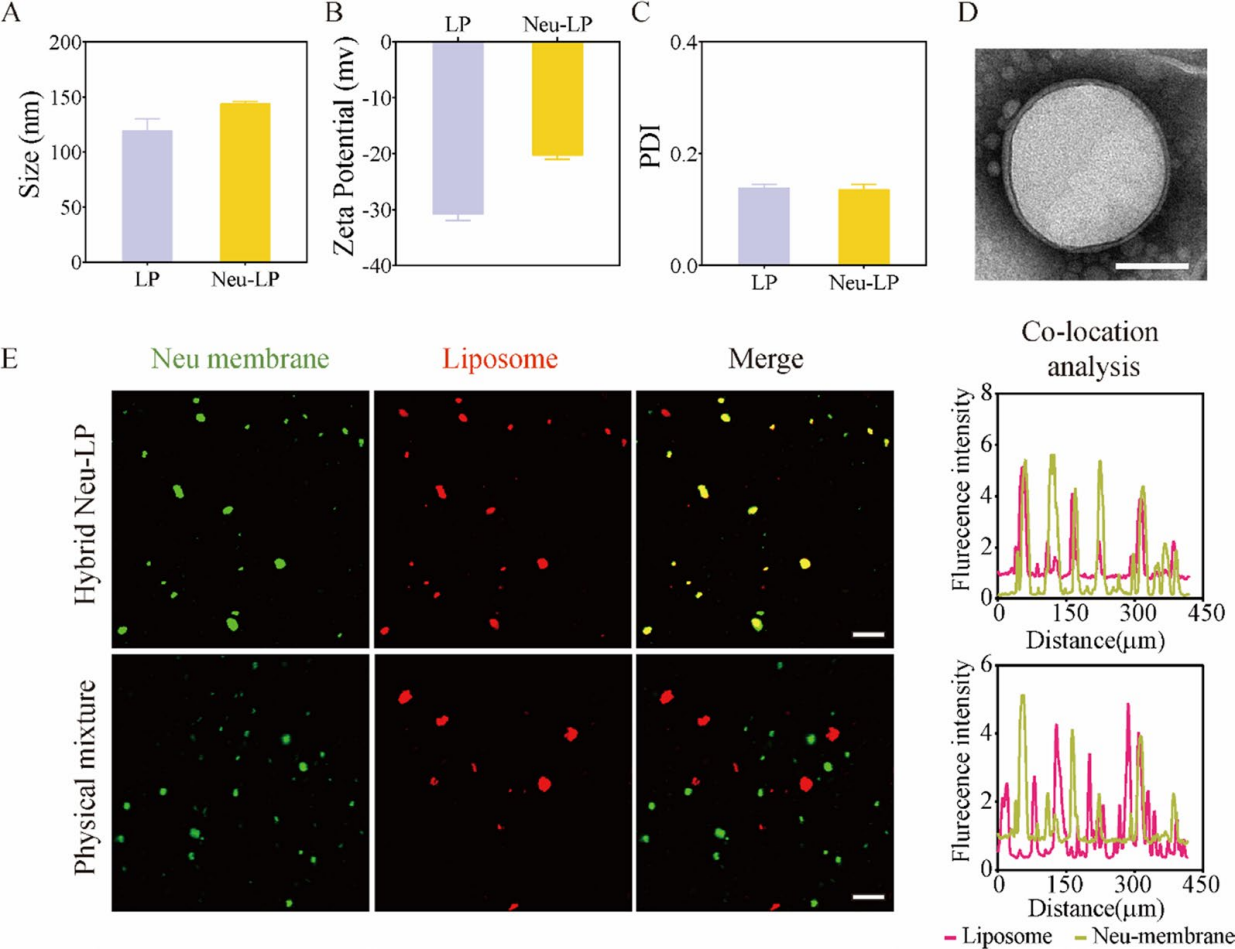
Fabrication of Neu-LPs. **A**–**C** DLS measurements of Neu-LP size (**A**), zeta potential (**B**), and polydispersity index (**C**) (*n* = 3). **D** Representative TEM image of Neu-LPs stained with uranyl acetate. Scale bars, 50 nm. **E** Confocal fluorescence microscopy images and co-localization analysis of Neu-LPs (top) and a physical mixture of LPs and NMVs (bottom). Green, NMVs; red, LPs; scale bars, 500 nm. Statistical analysis was performed using Student’s *t*-test. Data are presented as mean ± SD

To synthesize Neu-LPs, LPs were prepared by thin-film hydration and then fused with NMVs by extrusion. As shown in Fig. 1A–C, the hydrodynamic size of Neu-LPs was 143.6 ± 2.1 nm and their zeta potential was − 20.2 ± 0.8 mV. Dynamic light scattering (DLS) results illustrate that the diameter of the unmodified LPs increased by 23 nm and their surface zeta potential became less negative after fusion with NMVs. Both Neu-LPs and LPs displayed a narrow size distribution (Additional file 1: Fig. S1A). Transmission electron microscopy (TEM) revealed that Neu-LPs exhibited a spherical shape commonly observed for conventional liposomes (Fig. 1D and Additional file 1: Fig. S1B). Also, we investigated the stability of Neu-LPs by suspended them in water. The results demonstrated that Neu-LP had a similar stability as LP, as the sizes of Neu-LP remained stable throughout 7 days (Additional file 1: Fig. S2). Confocal fluorescence microscopy was then used to examine the fusion of LPs and NMVs. LPs were labeled with a red fluorescent dye and fused with NMVs labeled with a green fluorescent dye. After extrusion, Neu-LPs showed obvious dye colocalization, while a physical mixture of LPs and NMVs displayed distinct green and red fluorescent puncta (Fig. 1E).

### Protein composition analysis of Neu-LPs

**Fig. 2:**
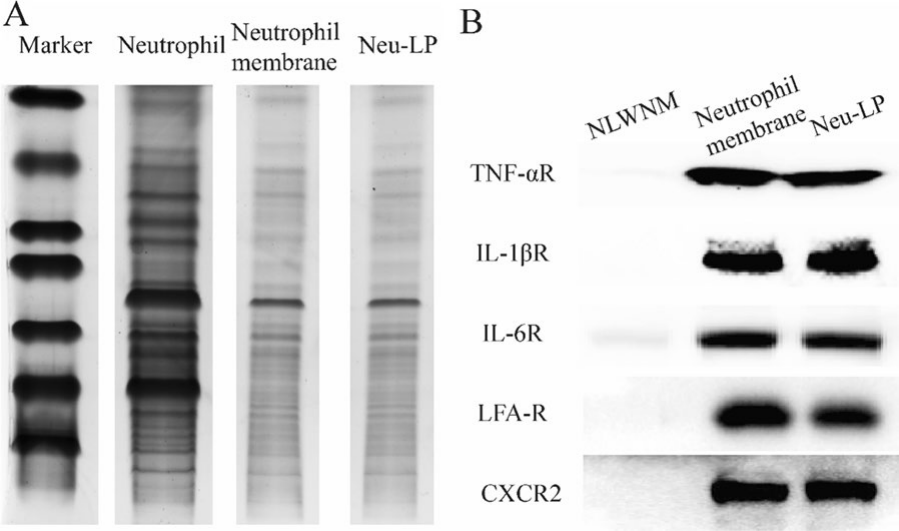
Membrane protein composition of Neu-LPs. **A** SDS-PAGE analysis of neutrophils, NMVs, and Neu-LPs. **B** Western blotting analysis of characteristic protein bands (TNFαR, IL1βR, IL6R, LFA-R, and CXCR2) in NMVs and Neu-LPs. NLWNM: Neutrophil lysate without neutrophil membrane protein

The protein composition of Neu-LPs was determined by sodium dodecyl sulfate–polyacrylamide gel electrophoresis (SDS-PAGE). The protein profile of NMVs was mostly preserved in Neu-LPs (Fig. 2A). Moreover, the presence of critical receptor proteins responsible for cytokine binding, including TNFα receptor (TNFαR), IL1β receptor (IL1βR), IL6 receptor (IL6R), lymphocyte function-associated antigen receptor (LFA-R), and C-X-C motif chemokine receptor (CXCR2), on Neu-LPs was confirmed by western blot analysis (Fig. 2B). These results confirmed the successful transfer of neutrophil membrane proteins into Neu-LPs.

### Inflammatory cytokine-binding ability of Neu-LPs

**Fig. 3:**
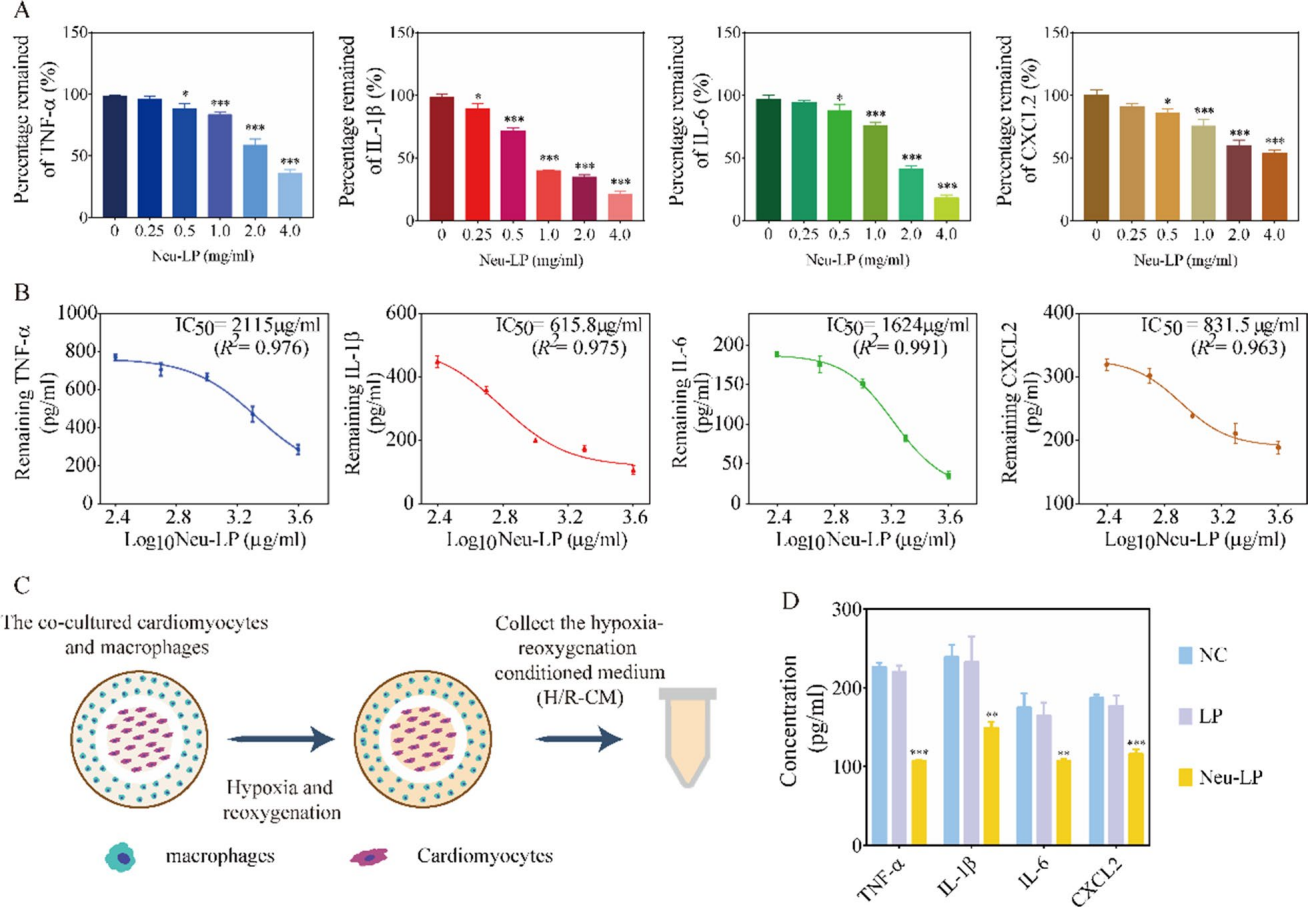
Inflammatory cytokine binding ability of Neu-LPs in vitro. **A** Neutralization dose response of proinflammatory factors (TNFα, IL1β, IL6, and CXCL2) by Neu-LPs. *P < 0.05 and ***P < 0.001 compared with the 0 mg/ml group. **B** Binding capacity of Neu-LPs to inflammatory cytokines (TNFα, IL1β, IL6, and CXCL2). IC_50_ values were derived from the variable slope model using GraphPad Prism 7. **C** Schematic illustration of a direct co-culture of macrophages and cardiomyocytes. **D** Expression levels of proinflammatory cytokines (TNFα, IL1β, IL6, and CXCL2) in the in vitro myocardial inflammation model with or without Neu-LP treatment. Statistical analysis was performed using one-way ANOVA. Data are presented as mean ± SD. **P < 0.01 and ***P < 0.001 compared with the NC group

Next, we tested the inflammatory cytokine- or chemokine-binding ability of Neu-LPs. The proinflammatory cytokines TNFα, IL1β, IL6, and CXCL2 are the cytokines most commonly associated with the remodeling process post-MI [20, 24]. These representative cytokines and chemokines were incubated with various doses of Neu-LPs, and the dose-dependent sequestration profiles of Neu-LPs were observed (Fig. 3A). Neu-LPs had a half maximal inhibitory concentration (IC_50_) value of 2115 μg/mL for TNFα binding, 615.8 μg/mL for IL1β binding, 1624 μg/mL for IL6 binding, and 831.5 μg/mL for CXCL2 binding (Fig. 3B). Benefiting from their abundant cytokine receptors, Neu-LPs could absorb those proinflammatory cytokines with the concentration increased, and no such results were observed when incubated with LPs. LPs demonstrated no specific naturalization ability and binding capacity of cytokines even with highest concentration (Additional file 1: Fig. S3). These results demonstrate that Neu-LPs may sequester proinflammatory cytokines and chemokines, leading to effective inhibition of activated inflammatory states. While this study involves the representative factors TNFα, IL1β, IL6, and CXCL2, the cytokine neutralization capability of Neu-LPs is also applicable to many other types of cytokines due to the abundant cytokine receptors on their membranes.

Ischemia leads to death of cardiomyocytes and stimulation of inflammatory cells, triggering activation of an intense inflammatory reaction [8, 20]. In this study, we used an in vitro myocardial inflammation model to mimic the ischemia/reperfusion- induced inflammatory state (Fig. 3C). In this model, cardiomyocytes (H9C2 cells) and macrophages (RAW 264.7 cells) were co-cultured under hypoxia-reoxygenation (H/R) conditions. This led to a significant increase in the levels of inflammatory cytokines (TNFα, IL1β, IL6, and CXCL2), confirming activation of inflammatory responses in this model. Next, we tested the cytokine binding performance of Neu-LPs by incubating them with conditioned medium from the H/R co-culture model (H/R-CM). We found that the levels of TNFα, IL1β, IL6, and CXCL2 were significantly decreased in H/R-CM after treatment with Neu-LPs (Fig. 3D). This result suggests that Neu-LPs could effectively neutralize cytokines and thus ameliorate inflammation in this in vitro myocardial inflammation model.

### Cardioprotective effect of Neu-LPs in vitro

**Fig. 4:**
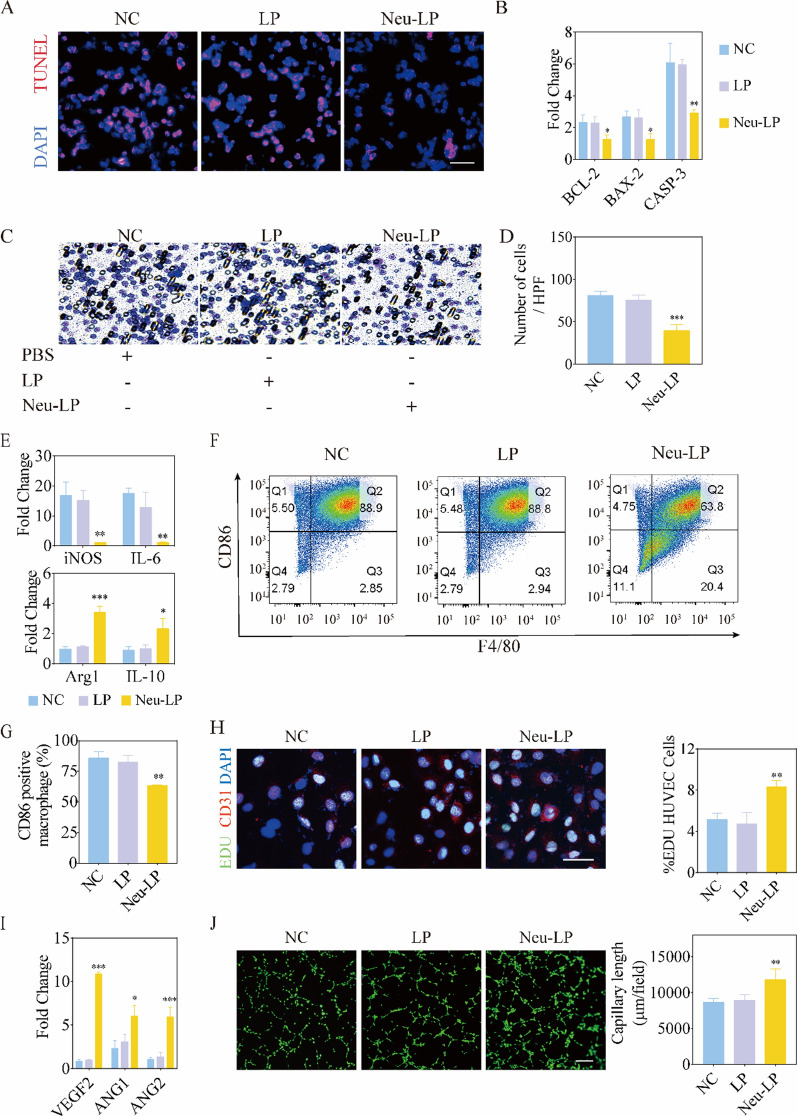
Cardioprotective and immunoregulatory effects of Neu-LPs in vitro. **A** Representative TUNEL staining of inflammation-stimulated cardiomyocytes treated with or without Neu-LPs. **B** Quantitative PCR analysis of apoptosis genes in cardiomyocytes after treatment with Neu-LPs (*n* = 3). **C**, **D** Microscopy images (**C**) and quantitative analysis (**D**) of neutrophil chemotaxis after treatment with Neu-LPs (*n* = 3). **E** Expression levels of M1 and M2 macrophage markers following treatment with Neu-LPs. **F**, **G** Representative flow cytometry graphs (**F**) and quantitative analysis (**G**) of macrophage polarization after treatment with Neu-LPs (*n* = 3). **H** Representative fluorescence images and quantitative analysis of an EdU assay for endothelial cell proliferation rate (*n* = 3). Scale bars, 50 μm. **I** Quantitative PCR of proliferation genes in HUVECs after treatment with Neu-LPs (*n* = 3). **J** Representative fluorescence images and quantitative analysis of tube formation by HUVECs after treatment with Neu-LPs (*n* = 4). Scale bars, 100 μm. Statistical analysis was performed using one-way ANOVA. Data are presented as mean ± SD. NC, negative control group which is treated with PBS. *P < 0.05, **P < 0.01, and ***P < 0.001 compared with the NC group

To evaluate the protective effect of Neu-LPs on cardiomyocytes from binding inflammatory cytokines, H9C2 cells were seeded in supplemented media mixed with H/R-CM in a 1:1 ratio and incubated with PBS, LPs, or Neu-LPs. TUNEL assay results show that Neu-LP treatment effectively inhibited apoptosis of the cardiomyocytes in the inflammatory medium (Fig. 4A). Additionally, the expressions of apoptotic genes were significantly decreased in the Neu-LPs group (Fig. 4B). All these results suggest that Neu-LPs could protect cardiomyocytes against H/R inflammatory injury by adsorbing cytokines.

### Effect of Neu-LPs on neutrophil chemotaxis in vitro

Cytokines and chemokines often have the unique ability to self-amplify by directing the recruitment of inflammatory cells to the injury site, which in turn provide an additional source of local cytokine production and amplification of the local inflammatory response [25]. Excessive inflammatory cell infiltration may exacerbate myocardial injury by prolonging the proinflammatory response. To further examine the effect of Neu-LPs on inflammatory cell infiltration, a transwell model was employed. Neutrophils were seeded in the upper chamber while H/R-CM was added to the lower chamber with PBS, LPs, or Neu-LPs. Compared with the LPs group, Neu-LPs reduced inflammatory cell migration, which might be due to the chemokine neutralization (Fig. 4C and D).

### Effect of Neu-LPs on macrophage polarization

Monocytes play an important role in the inflammatory response and reparative phase following MI [26]. After MI, monocytes are recruited to the injured myocardium where they differentiate into M1 macrophages during the initial proinflammatory phase and then switch their phenotype to anti-inflammatory M2 macrophages in the reparative phase [27, 28]. The prolonged presence of M1 macrophages can extend the proinflammatory phase and cause expansion of the infarcted area, thereby delaying the reparative phase and formation of scar tissue mediated by M2 macrophages and exacerbating adverse left ventricular remodeling. Meanwhile, macrophage polarization can be influenced by cytokines and chemokines in the microenvironment. To evaluate the effect of Neu-LPs on macrophage polarization, RAW 264.7 cells maintained in an unpolarized state were seeded in supplemented media mixed with H/R-CM in a 1:1 ratio and incubated with PBS, LPs, or Neu-LPs. Then, the gene expressions of M1 and M2 markers were investigated using polymerase chain reaction (PCR) assays (Fig. 4E). After Neu-LP treatment, the expressions of M1 markers [inducible nitric oxide synthase (iNOS) and IL6] were significantly decreased, while the expressions of M2 markers [arginase 1 (Arg1) and IL10] were increased, indicating that Neu-LPs inhibited M1-like macrophage activity and allowed a switch to an M2 phenotype. By contrast, LPs did not affect the macrophage state and the macrophages maintained their proinflammatory phenotype induced by H/R-CM. Fluorescence-activated cell sorting analyses also showed a similar result (Fig. 4F and G). The macrophage phenotype changed from proinflammatory to anti-inflammatory, as judged by the decrease in CD86 expression (a surface marker for proinflammatory macrophages). These findings validate the immunomodulatory capacity of Neu-LPs on macrophages: Neu-LPs regulated macrophage polarization and induced a switch to an anti-inflammatory and reparative state.

Macrophages have been widely proven to participate in both the processes of repair and angiogenesis. Therefore, we evaluated the angiogenesis promotion effect of Neu-LPs caused by their modulation of macrophage phenotype in vitro. Human umbilical vein endothelial cells (HUVECs) and RAW 264.7 cells were co-cultured using a transwell system. HUVECs were seeded in the bottom chamber of the transwell, while RAW 264.7 cells were added to the upper chamber in supplemented media mixed with H/R-CM in a 1:1 ratio and incubated with LPs or Neu-LPs. Treatment with Neu-LPs significantly promoted HUVEC proliferation and angiogenesis, as revealed by EdU (Fig. 4H), PCR (Fig. 4I), and tube formation (Fig. 4J) assays. These results confirm the angiogenesis promotion effect of Neu-LPs by immunomodulation.

### In vitro migratory ability of Neu-LPs

**Fig. 5:**
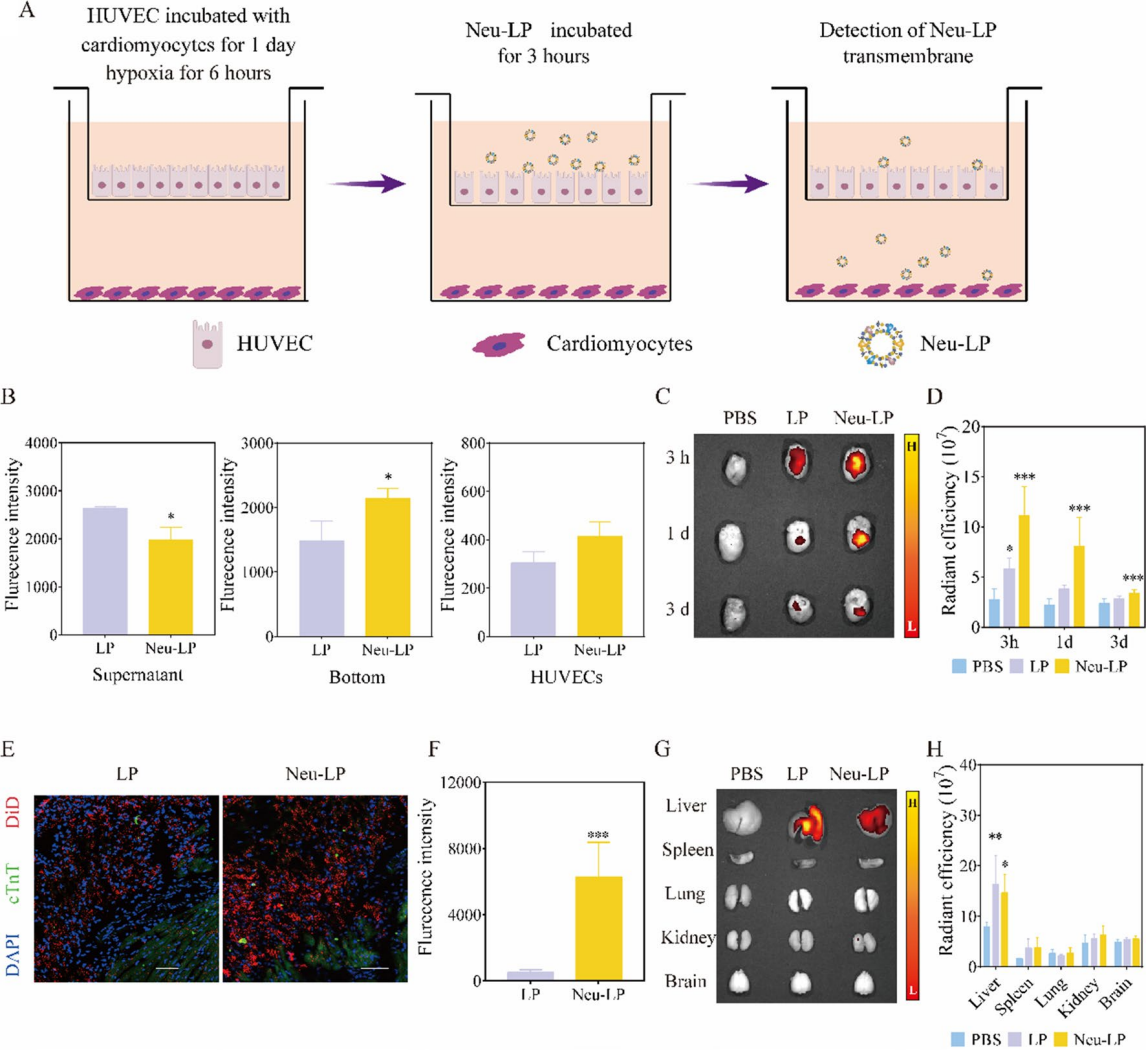
Biodistribution of Neu-LPs in vitro and in vivo. **A** Schematic illustration of the in vitro transmembrane model to evaluate the penetration and transport of Neu-LPs across a monolayer of HUVECs. **B** Quantification of Neu-LP distribution in the transwell system (*n* = 3). **C**, **D** Ex vivo fluorescence images (**C**) and quantification (**D**) of hearts from MI/R mice after treatment with Neu-LPs, LPs, or PBS at various timepoints (3 h, 1 day, and 3 days) (*n* = 6). *P < 0.05 and ***P < 0.001 compared with the PBS group. **E** Fluorescence microscopy images of Neu-LP distribution in infarcted areas. Scale bars, 100 μm. **F** Quantitative analysis of the fluorescence intensity of Neu-LPs (*n* = 6). ***P < 0.001 compared with the LPs group. **G**, **H** Representative fluorescence images (**G**) and quantitative analysis of fluorescence intensity (**H**) in major organs after intravenous injection of Neu-LPs (*n* = 6). *P < 0.05 and **P < 0.01 compared with the PBS group. Comparisons between two groups were performed with Student’s *t*-test. One-way analysis of variance (ANOVA) was employed for comparisons among more than two groups. Data are presented as mean ± SD

The migratory ability of Neu-LPs towards the inflammatory site in vitro was tested in a HUVECs and H9C2 cells coculture transwell system (Fig. 5A). After hypoxia stimulation for 6 h, DiD-labeled Neu-LPs or LPs were added to the upper chamber for 3 h. Particle distribution was evaluated in three compartments: free particles in the upper chamber, entrapped particles in the endothelial monolayer, and transported particles in the lower chamber. As shown in Fig. 5B, the proportion of Neu-LPs recovered from the bottom chamber was 47.3%, which was 1.4-fold higher than that of LPs. A relative decrease in the proportion of Neu-LPs found in the upper chamber accompanied this difference. Meanwhile, Neu-LP binding to inflamed endothelial cells was also higher than that of LPs. This result shows that, under inflammatory conditions, neutrophil membrane decoration could increase particle transport through an endothelium monolayer, thereby increasing the targeting ability of Neu-LPs towards ischemic myocardium compared with LPs.

### In vivo targeting ability of Neu-LPs

Next, the in vivo targeting ability of Neu-LPs was explored in an MI/R model using ex vivo imaging. Successful acute MI/R was confirmed by ST-segment-characterized echocardiograph (Additional file 1: Fig. S4). One day post reperfusion, mice were intravenously injected with PBS, LPs, or Neu-LPs. As shown in Fig. 5C and D compared with the LPs group, the fluorescence intensity in the infarcted area of the Neu-LPs group was significantly higher at all timepoints post administration, which ranged from 3 h to 3 days (*P* < 0.05, *n* = 6), suggesting that Neu-LPs could identify chemotactic signals and recruit into the infarcted area. Histological sections were then taken to evaluate the homing efficiency of Neu-LPs and obtain more details on their targeting to infarcted regions in vivo. As shown in Fig. 5E and F; Additional file 1: Fig. S5, the homing ability of Neu-LPs to ischemic myocardium was significantly higher than that of LPs, and Neu-LPs specifically distributed in damaged myocardium rather than normal myocardial tissue. In contrast to this enhanced accumulation in injured myocardium, lower accumulation of Neu-LPs was noted in the liver (Fig. 5G and H), which might be due to the presence of self-tolerance proteins and the good immune evasion of NMVs, as was previously reported [29].

### Cytokine neutralization and immunomodulatory effects of Neu-LPs in vivo

**Fig. 6:**
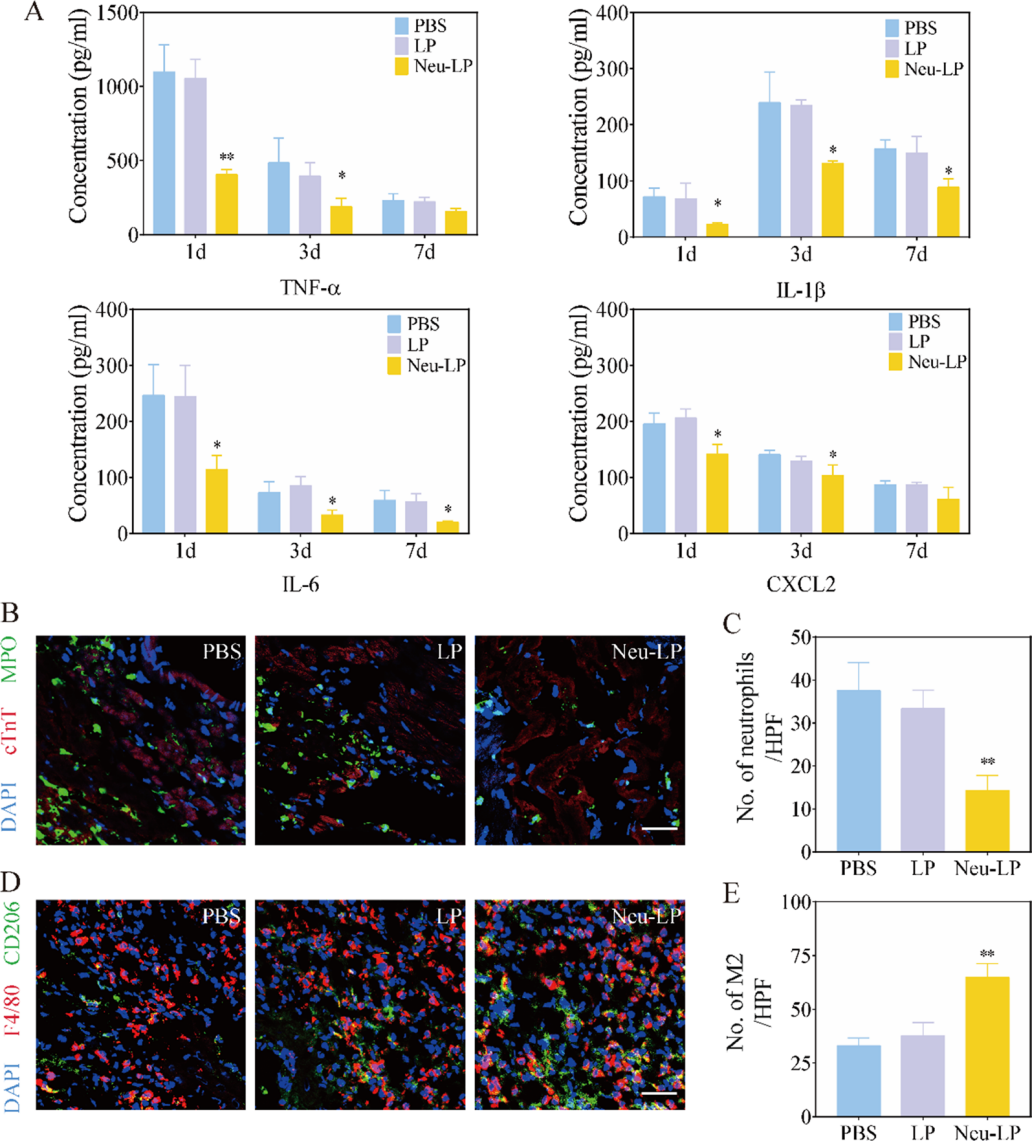
In vivo immunoregulation ability of Neu-LPs. **A** Concentration profiles of inflammatory cytokines (TNFα, IL1βand IL6) and chemokine (CXCL2) in ischemic hearts at various timepoints (1, 3, and 7 days) following injection of Neu-LPs (*n* = 3). **B** Infiltration of neutrophils in the infarcted area three days after Neu-LP treatment detected by myeloperoxidase (MPO) (*n* = 3). Scale bars, 100 μm. **C** Quantitative analysis of MPO-positive cells. **D**, **E** Fluorescence images (**D**) and quantitation (**E**) of macrophages (F4/80, red) and M2 macrophages (CD206, green) in heart tissue (*n* = 3) three days after Neu-LP treatment. Scale bars, 100 μm. Statistical analysis was performed using one-way ANOVA. Data are presented as mean ± SD. *P < 0.05 and **P < 0.01 compared with the PBS group

The in vivo cytokine neutralization and immunomodulatory effects of Neu-LPs were further evaluated in the mouse model of MI/R. Enzyme-linked immunosorbent assay (ELISA) analysis confirmed a significant decrease in the expression levels of proinflammatory factors (TNFα, IL1β, and IL6) and chemokine (CXCL2) in ischemic regions at various time points (1, 3, and 7 days) after Neu-LP administration (Fig. 6A). Histological analysis also illustrated a reduction in immune cell infiltration in mice who received Neu-LPs. Three days after treatment, the Neu-LPs group displayed decreased infiltration of neutrophils in the peri-infarct region compared to other groups (Fig. 6B and C). Meanwhile, Neu-LPs promoted a shift in macrophage phenotype to an anti-inflammatory subtype (M2) at 3 days post administration, and the number of M2 cells in the peri-infarct region increased significantly compared to other groups (Fig. 6D and E). Meanwhile, we detected the macrophage recruitment by flow cytometry at day 3 after treatment. We found that the macrophage recruitment in Neu-LPs group was 52.6%, which was decreased by 27.3% and 23.3% compared with that of the PBS and LP groups (P < 0.01). And M2 in the Neu-LPs group was increased to 46.0%, which was much higher than that in PBS group (20.0%) and LP group (24.0%) (P < 0.01) (Additional file 1: Fig. S6). These results suggest that Neu-LPs could neutralize cytokine release from injured myocardium, alleviate inflammation, and shift the balance towards the reparative process.

### Repair promotion and cardiac protection ability of Neu-LPs

**Fig. 7:**
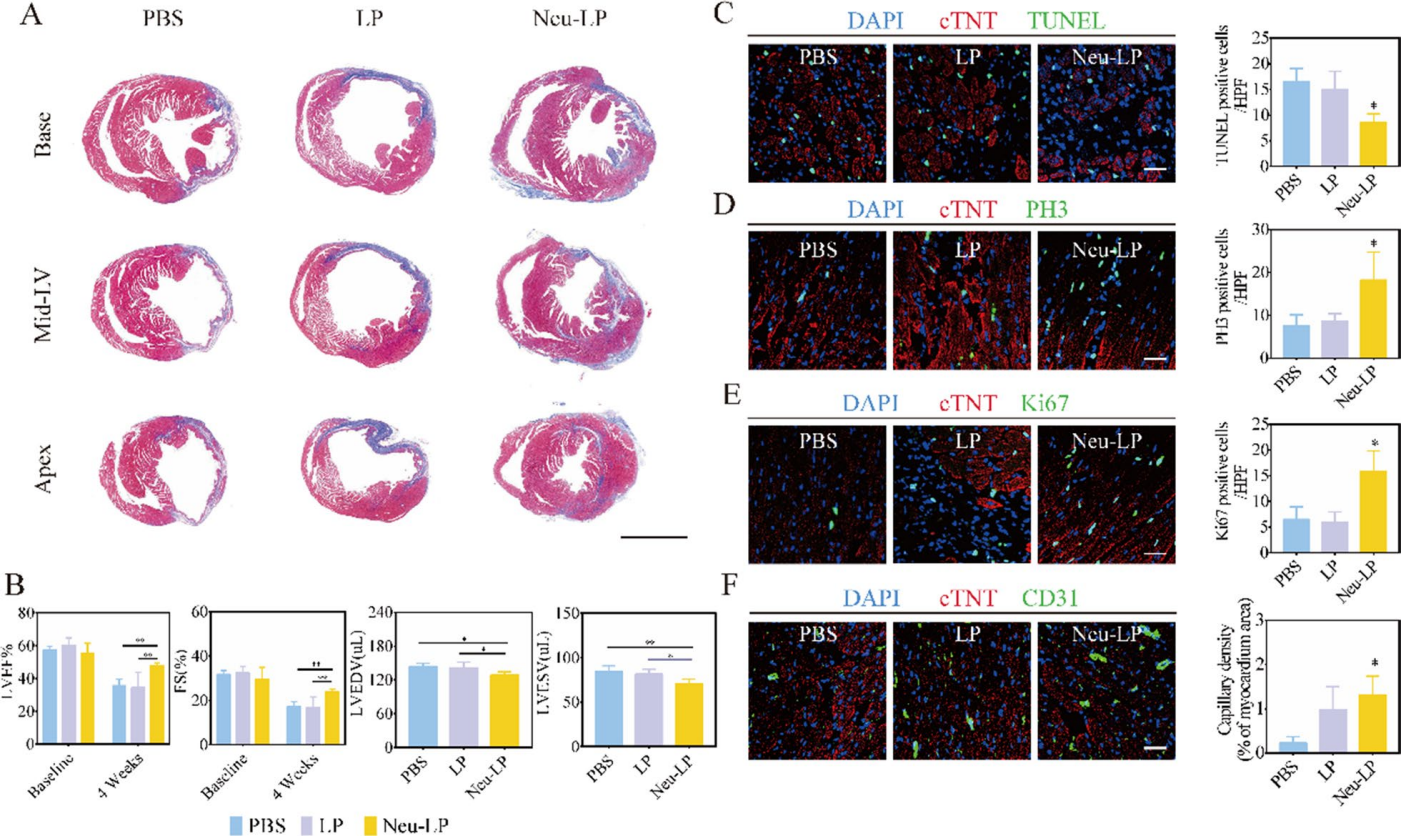
Cardiac repair effects of Neu-LPs in vivo. **A** Representative Masson’s trichrome staining of the base, mid-left ventricular (LV), and apical regions of an infarcted heart 4 weeks after treatment with Neu-LPs. Scale bars, 2.5 mm. **B** LVEF, FS, LVEDV, and LVESV values measured by echocardiography at 4 weeks (*n* = 6 animals per group). **C** Representative TUNEL staining images and quantification of TUNEL-positive apoptotic cells in infarcted regions 7 days after MI (*n* = 3). Scale bars, 100 μm. **D** Representative confocal images and quantitative analysis of PH3-positive nuclei in the peri-infarct region 4 weeks after treatment with Neu-LPs (*n* = 3). Scale bars, 100 μm. **E** Representative images and quantification of Ki67-positive cells at 7 days post-treatment with Neu-LPs (*n* = 3). Scale bars, 100 μm. **F** Representative micrographs of CD31-positive blood vessels and quantification of capillary density in hearts 4 weeks after treatment with Neu-LPs (*n* = 3). Scale bars, 100 μm. Statistical analysis was performed using one-way ANOVA. Data are presented as mean ± SD. *P < 0.05 and **P < 0.01 compared with the PBS group

Next, we assessed the therapeutic efficiency of Neu-LPs. The histology of injured heart was evaluated by hemotoxylin-eosin (HE) and Masson trichrome staining at day 3 after Neu-LP treatment. The results illustrated that Neu-LPs decreased the infiltration of inflammatory cell and reduced myocardial fibrosis at early time points (Additional file 1: Fig. S7). Masson trichrome staining after 4 weeks results displayed apparent heart morphology protection with the highest amount of viable myocardium in the Neu-LPs group compared with the LPs and PBS groups (Fig. 7A). As indicators of cardiac function, left ventricular ejection fraction (LVEF) and fractional shortening (FS) were also measured at baseline (4 h post reperfusion), post-treatment 1, 10 and 28 days (Additional file 1: Fig. S8) There was no significant difference of LVEF or FS among the three groups, and the values of LVEF and FS were significantly decreased at day 1. However, the LVEF was increased in the mice treated with Neu-LPs compared with PBS group and LP group at day 10. The FS showed a similar trend as LVEF. With the time developed, the LVEF and FS of Neu-LP group were higher than PBS group or LP group at day 28. The cardiac function remained at low level post MI/R injury in the PBS and LP treated groups within 4 weeks. In addition, Neu-LP treatment led to a reduction in left ventricular end-diastolic volume (LVEDV) and end-systolic volume (LVESV) (Fig. 7B). These compounding results demonstrate the therapeutic benefits of Neu-LPs in acute MI.

We also found that TUNEL-positive cells were significantly reduced in the infarct region of the Neu-LPs group compared with other groups (Fig. 7C). Additionally, the number of phospho-histone H3 (PH3)-positive cells and Ki67-positive cells in the peri-infarct region of the Neu-LPs group were significantly higher than those of other groups (Fig. 7D and E). Furthermore, treatment with Neu-LPs increased the regeneration of CD31-positive vasculature in the infarcted myocardium (Fig. 7F). Taken together, these results indicate that Neu-LPs could reduce cell apoptosis, promote cell proliferation, and promote angiogenesis in infarcted hearts, which might contribute to their enhanced cardiac reparative ability.

### Safety evaluation of Neu-LPs

**Fig. 8:**
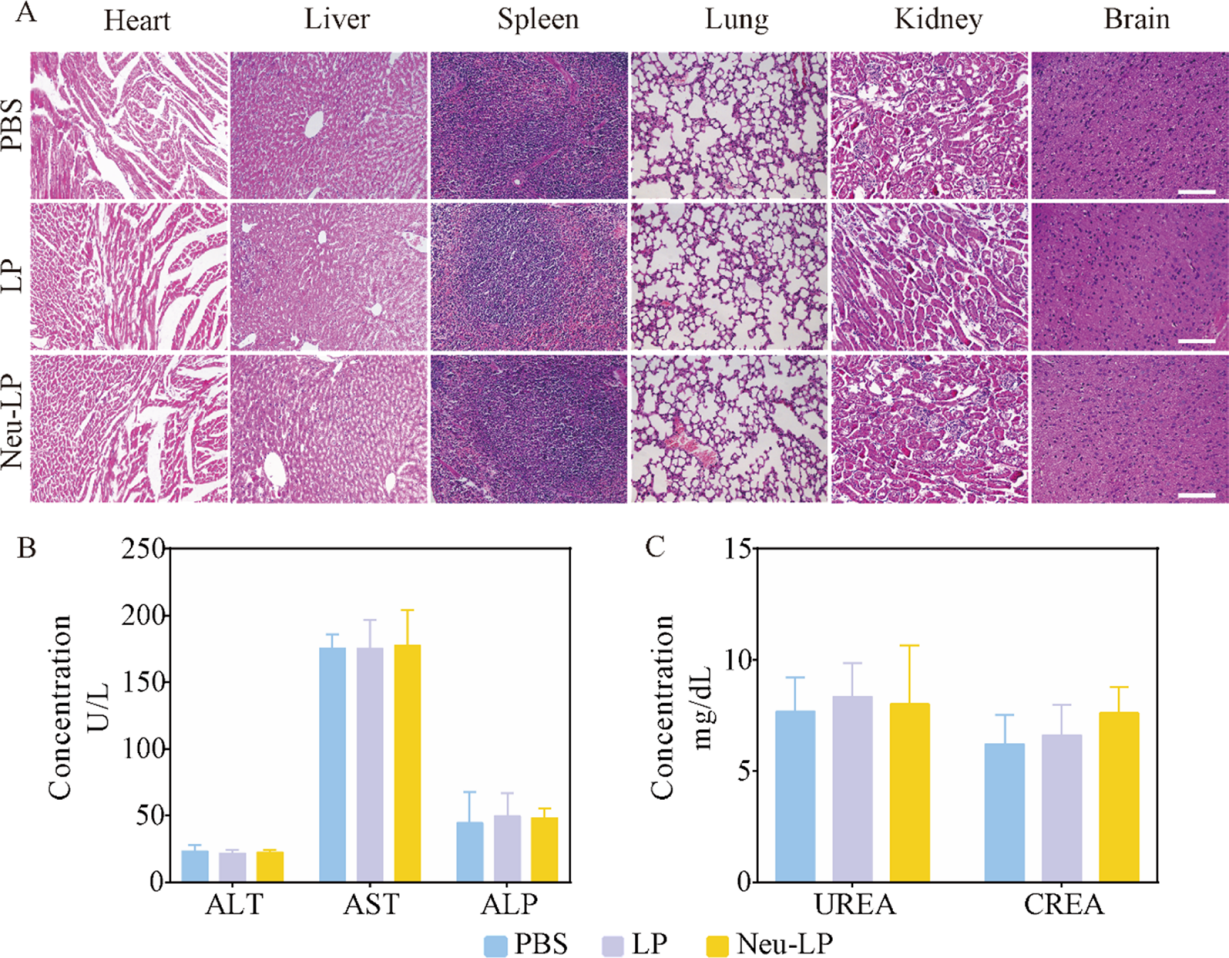
Safety evaluation of Neu-LPs. **A** H&E staining of the major organs (*n* = 6). Scale bars, 50 μm. **B**, **C** Quantitative analysis of blood biochemistry results indicating liver (**B**) and kidney (**C**) function from peripheral blood samples. Statistical analysis was performed using one-way ANOVA. Data are presented as mean ± SD

To evaluate the safety of Neu-LPs, healthy ICR mice were intravenously injected with Neu-LPs, LPs, or PBS at a dose of 20 mg/kg every other day for one week (*n* = 6). Hematoxylin and eosin (H&E) staining of the major organs (including the heart, liver, spleen, lung, kidney, and brain) revealed no pathological changes in the Neu-LPs and LPs groups relative to the PBS group (Fig. 8A). Liver and kidney function assays were also performed 1 day after the final injection. There were no significant changes in serum levels of liver (ALT, AST, and ALP) and kidney (UREA and CREA) function markers between the Neu-LPs or LPs and PBS groups (Fig. 8B and C). To further assess the immunogenicity of Neu-LP, we detected the expression level of IgM in the serum after injection of Neu-LPs. The results showed that serum levels of IgM had no significant differences among three groups and Neu-LPs do not cause significant acute immune responses in C57 mice (Additional file 1: Fig. S9).These results indicate that Neu-LPs possess a good safety profile, suggesting their potential use in treating cardiovascular diseases.

## Conclusion

In this study, we generated neutrophil-mimetic liposomes, Neu-LPs, and demonstrated their potential as a broad-spectrum anti-inflammatory agent for MI management. Neu-LPs contained their parent cell properties and inherited inflammation-targeting and cytokine-binding capacities. Due to their abundant chemokine and cytokine receptors, Neu-LPs accumulated in infarcted hearts in response to inflammatory signals and efficiently neutralized inflammatory cytokines, such as TNFα, IL1β, IL6, and CXCL2. In a mouse model of MI/R, Neu-LPs inhibited the intense inflammation, regulated the immune microenvironment to a reparative phenotype, and induced therapeutic cardiac repair by suppressing cardiac injury and promoting angiogenesis. Unlike existing anti-cytokine agents that inhibit single targets, Neu-LPs provided an inflammation-targeted and broad-spectrum blockade that restrained the inflammation cascade during MI. Many other diseases, such as myocarditis, ischemia–reperfusion injury of the brain and kidney, are also marked by inflammatory responses that can damage host tissue and cause organ dysfunction. Neu-LPs present a promising anti-inflammatory strategy for treatment of these diseases that could eventually improve clinical outcomes in patients. Neu-LPs also exhibit high downstream translation potential for developing personalized nanomedicines through the use of established liposome technologies and natural cell membranes. Finally, this membrane fusion approach could be extended to other immune cells and could be combined with other agents to develop targeted and synergistic therapies for MI and various inflammation-related diseases.

## Experimental section

### Materials

1,2-Dioleoyl-*sn*-glycero-3-phosphocholine (DOPC) was purchased from Avanti Polar Lipids Pharmaceutical Co., Ltd (Shanghai, China). 1,2-distearoyl-*sn*-glycero-3-phosphoethanolamine-N-[maleimide(polyethylene glycol)] (DSPE-PEG-Mal, 3400 Da) was purchased from Xi’an Ruixi Biotech Co., Ltd (Xian, China). 1,19-Dioctadecyl-3,3,39,39-tetramethylindodicarbocyanine perchlorate (DiD) was supplied by Sigma-Aldrich (Carlsbad, CA, USA). 1,1′-Dioctadecyl-3,3,3′,3′-tetramethylindocarbocyanine perchlorate (DiI) was obtained from Invitrogen (Carlsbad, CA, USA). 4′,6-Diamidino-2-phenylindole (DAPI, C1002) was obtained from Beyotime Biotechnology Co., Ltd (Nantong, China). Accustain Trichrome Stain (Masson) kit and Percoll were obtained from Sigma-Aldrich. Matrigel was obtained from Corning (Corning, NY, USA). Recombinant human TNFα (ab259410) and ELISA kits for TNFα (ab208348), IL1β (ab197742), IL6 (ab222503), and CXCL2 (ab184862) were purchased from Abcam (Cambridge, MA, USA). BCA Protein Assay Kit (P0010) was purchased from Beyotime Biotechnology. TRIzol reagent and RT-PCR Kit were obtained from Invitrogen. Anti-CD34 antibody (sc-53511) was purchased from Santa Cruz Biotechnology (Santa Cruz, CA, USA). Anti-TNFα antibody (ab183218), anti-IL1R antibody (ab106278), anti-IL6R antibody (ab222101), anti-LFA1 antibody (ab13219), and anti-CXCR2 antibody (ab225732) were purchased from Abcam. Anti-β-actin antibody (AA128), HRP-labeled goat/anti-mouse IgG (H + L, A0216) and HRP-labeled goat/anti-rabbit IgG (H + L, A0208) were obtained from Beyotime Biotechnology. Anti-PH3 antibody (ab321073), anti-Ki67 antibody (ab16667), anti-CD31 antibody (ab222783), anti-F4/80 antibody (ab6640), anti-CD206 antibody (ab125028), and anti-cardiac troponin T antibody (ab8295) were all obtained from Abcam.

### Animal

Male C57BL/6 mice (aged 8–12 weeks, 20–25 g in weight) and male ICR mice (aged 8–12 weeks, 30–35 g in weight) were purchased from Shanghai SLAC Laboratory Animal Ltd. All animal experimental procedures were approved by the Animal Care and Use Committee of Zhongshan Hospital, Shanghai, People’s Republic of China and were in compliance with the Guide for the Care and Use of Laboratory Animals published by the National Research Council (US) Institute for Laboratory Animal Research.

### Neutrophil collection

Fresh human peripheral blood neutrophils were obtained from Zhongshan Hospital. Neutrophil isolation was performed according to previous studies with some modifications [30]. Briefly, neutrophils were purified by density gradient centrifugation. Leukocyte-rich plasma was placed over a three-layer Percoll gradient of 78%, 69%, and 52% and centrifuged at 1000×*g* for 30 min. The interface containing neutrophils was between the 69% and 78% gradient layers and the upper part of the 78% layer. The isolated neutrophils were rinsed with PBS, suspended in serum-free RPMI media, and then stimulated with recombinant human TNFα (50 ng/mL) for 2 h at 37 °C. Next, the stimulated cells were resuspended in serum-free RPMI and cryopreservation medium and stored at − 80 °C.

### Neutrophil membrane derivation

First, neutrophils were suspended in lysing buffer containing 225 mM d-mannitol, 75 mM sucrose, 30 mM Tris–HCl (pH 7.5), 0.2 mM EGTA, and a protease and phosphatase inhibitor cocktail. Then, the cells were disrupted using a Dounce homogenizer with a tight-fitting pestle (20 passes). Next, the homogenized solution was centrifuged at 20,000×*g* for 30 min at 4 °C. Then, the supernatant was collected and centrifuged at 100,000×*g* for 40 min at 4 °C. Subsequently, membranes were collected as sedimentation at the bottom of the centrifuge tube. Membrane content was quantified using a BCA kit. Approximately 100–200 million neutrophils generated 1 mg of membrane protein. Neutrophil membrane was suspended in 0.2 mM EDTA to a protein concentration of 2 mg/mL and stored at − 80 °C for subsequent studies.

### Synthesis of nanoparticles

Neu-LPs were synthesized via a lipid film rehydration process [14]. Briefly, 9 mg of DOPC and 1 mg of DSPE-PEG-Mal were dissolved in 10 mL of chloroform and dried in a flask by rotary evaporation for 1 h. Then, the thin lipid film was hydrated with 10 mL of distilled water at 37 °C for 15 min and sonicated at a frequency of 52 kHz and a power of 100 W for 30 min. For membrane coating, the neutrophil membrane was mixed with the liposome cores at a weight ratio of 30:1 (polymer-to-membrane protein). The mixture was then sonicated with a bath sonicator for 10 min. The particles were then extruded through membranes with pore sizes of 400, 200, and 100 nm using an extruder. Fluorescent dye-labeled Neu-LPs were prepared by the same method as Neu-LPs except 25 μg of DiD (Neu-LPs-DiD) were added to the lipid solution. Then free unlabeled fluorescence DiD was separated by centrifugation at 100,000*g* for 30 min and determined by using a microplate reader (SpectraMax^®^ M5, Molecular Devices). The fluorescence label efficiency was calculated as follows:

$$\mathrm{Label \, efficiency }=\frac{\mathrm{Total \,amount }-\mathrm{ The \,amount \,in \,supernatant}}{\mathrm{Total \,amount }}\times 100\mathrm{\%}$$

The DiD label efficiency of Neu-LP was 91.2 ± 1.4%.

### Nanoparticle characterization

The hydrodynamic diameters and zeta potentials of Neu-LPs were measured by DLS (Zetasizer Nano ZS, Malvern Instruments, Malvern, UK). The morphology of Neu-LPs was visualized by TEM (H-600, Hitachi, Tokyo, Japan). To evaluate the stability of Neu-LP, LP or Neu-LP were suspended in water at a concentration of 1 mg/mL. At set timepoints over the course of 7 days, the sizes of the samples were measured by DLS to test for aggregation.

### Membrane colocalization assay

A membrane colocalization study was performed as previously described with some modification [14]. NMVs were labeled with DiI (excitation/emission = 549/565 nm), and LPs were labeled with DiD (excitation/emission = 644/665 nm). Free dyes were removed by centrifugation at 100,000*g* for 30 min and the fluorescence label efficiency was calculated as mentioned above. The DiI label efficiency of NMV was 92.5 ± 2.2% and DiD label efficiency of LP was 93.2 ± 1.8%. Then, samples were prepared by extrusion or simple pipetting and thereafter were visualized under a confocal microscope (Leica Microsystems, Wetzlar, Germany).

### Cell culture

HUVECs, cardiomyocytes (H9C2), and macrophages (RAW 264.7) were purchased from American Type Culture Collection. HUVECs were cultured in human endothelial cell growth medium (ECM), while H9C2 cells and RAW 264.7 cells were cultured in high glucose culture medium. Cells were maintained at 37 °C in a 5% CO2 environment. All media were supplemented with 10% (v/v) FBS as well as penicillin and streptomycin (100 U/mL).

### Inflammatory factors neutralization assay

Proinflammatory factors (TNFα, I1β, IL-6, and CXCL2) were mixed with Neu-LPs to final concentrations of 0–4 mg/mL. The mixtures were incubated for 6 h at 37 °C and then centrifuged at 15,000×*g* for 20 min to remove the Neu-LPs. The cytokine concentrations in the supernatant were quantified by ELISA kits. Linear fitting of the proinflammatory factor curves were performed in GraphPad Prism 7.

### In vitro myocardial inflammation model

Macrophages (5 × 10^4^ cells) were seeded in the outer edges of a glass-bottom dish, while H9C2 cells (1 × 10^5^ cells) were seeded in the center region of the dish. Then, the culture chamber was continuously flushed with (5% CO_2_ and 95% N_2_) for 6 h at 37 °C to obtain an anoxic solution. The co-culture was then replaced in a CO_2_ incubator (5% CO_2_, 95% air, 37 °C) for reoxygenation. After this process, the levels of inflammatory cytokines in the hypoxia-reoxygenation conditioned medium (H/R-CM) were significantly increased [31]. The co-cultured cells were treated with supplemented media mixed with H/R-CM in a 1:1 ratio and incubated with PBS, LPs, or Neu-LPs for another 6 h. Changes in inflammatory cytokine (TNFα, IL1β, IL6, and CXCL2) levels were detected by ELISA assays.

### The TUNEL assay

H9C2 cells were seeded in 12-well plates (5 × 10^4^ cells per well) at 37 °C to permit cell adhesion and reach 80–90% confluence. The culture medium was replaced with supplemented RPMI media mixed with H/R-CM in a 1:1 ratio and the cells were incubated with PBS, LPs, or Neu-LPs. After 6 h of treatment, cardiomyocyte apoptosis rates were measured using a TUNEL Apoptosis Assay Kit and the cells were observed using an inverted fluorescence microscope (ZEISS Group)..

### Real-time PCR analysis

Changes in gene expression levels were determined by real-time PCR (RT-PCR). RNA was isolated from mouse cardiomyocytes, macrophages, endothelial cells, or ischemic myocardium tissue by TRIzol and reverse-transcribed into cDNA using the First-Strand Synthesis System for RT-PCR Kit. SYBR Green-based quantitative RT-PCR was performed using the Mx3000 Multiplex Quantitative PCR System. The relative expression was calculated using the comparative CT method (2^−ΔΔCt^). Triplicate samples were used for each experimental condition to determine relative expression levels. Primer sequences used are listed in Table 1.

**Table 1:** Primer sequences used for PCR analysis

Gene	Primer sequences
Actin	F: TCACCATGGATGATGATATCGC
	R: ATAGGAATCCTTCTGACCCATGC
TGF-β	F: CCACCTGCAAGACCATCGAC
	R: CTGGCGAGCCTTAGTTTGGAC
VEGF-A	F:AGGGCAGAATCATCACGAAGT
	R: AGGGTCTCGATTGGATGGCA
VEGF-2	F: CAAGTGGCTAAGGGCATGGA
	R: ATTTCAAAGGGAGGCGAGCA
Ang-1	F: AACCGGATTCAACATGGGCA
	R: TCTCACGACAGTTGCCATCG
Ang-2	F: CTGTTGAACCAAACAGCGGAG
	R: TCGAGAGGGAGTGTTCCAAGA
Bcl-2	F: CTTTGAGTTCGGTGGGGTCA
	R: GGGCCGTACAGTTCCACAAA
Bax-2	F: CATGGGCTGGACATTGGACT
	R: AAAGTAGGAGAGGAGGCCGT
Casp-3	F: GGCGCTCTGGTTTTCGTTAAT
	R: CCGAGATGTCATTCCAGTGCT
IL-10	F: AGCCTTATCGGAAATGATCCAGT
	R: GGCCTTGTAGACACCTTGGT

### Neutrophil chemotaxis assay

Neutrophils (1 × 10^4^ cells/well) were seeded in the upper chamber of a transwell system, while supplemented media mixed with H/R-CM in a 1:1 ratio and PBS, LPs, or Neu-LPs were added to the lower chamber. After incubation for 6 h, the chambers were removed. The cells were fixed with 4% paraformaldehyde and stained with 0.25% crystal violet for 10 min. After three rinses with PBS and air drying, the chambers were inverted on a glass slide and the number of chemotaxis-migrated neutrophils was counted under a microscope.

### Flow cytometric analysis

Cell suspensions of macrophages were stained with FITC-conjugated anti-F4/80 antibodies and PE-conjugated anti-CD86 antibodies (1:100 dilution) for 40 min at 4 °C. Flow cytometric analysis was performed on a FACSCalibur (BD Biosciences) and analyzed with FlowJo software (TreeStar, Inc., San Carlos, CA).

### Cell proliferation assay

EdU assays were performed to investigate cell proliferation. Briefly, HUVECs were seeded into the lower chamber of a transwell system, while RAW 264.7 cells were added to the upper chamber in supplemented media mixed with H/R-CM in a 1:1 ratio and incubated with PBS, LPs, or Neu-LPs. After 6 h of treatment, HUVECs were stained with anti-CD31 antibody and EdU-488 kit according to the manufacturer's protocol and then observed under an inverted fluorescence microscope. The number of positive cells was analyzed by ImageJ (US National Institutes of Health).

### Endothelial tube formation assay

An endothelial tube formation assay was performed as previously described with some modification [32]. Matrigel (200 μL) was first added to each well of a 48-well plate to evenly cover the bottom, and then the plate was placed in an incubator at 37 °C for 2 h until the Matrigel solidified. HUVECs (2 × 10^4^ cells/well) were seeded in the prepared 48-well plates and incubated at 37 °C for 4 h. Tube formation was observed under an inverted fluorescence microscope and capillary length was analyzed using ImageJ.

### Assay for chemotactic migration across a vascular barrier

An in vitro vascular intimal barrier model was constructed with HUVECs using a transwell cell culture system. Briefly, HUVECs (1 × 10^5^ cells/well) were seeded in the upper chamber and H9C2 cells (1 × 10^5^ cells/well) were added to the bottom chamber and cultured in medium containing 10% (v/v) FBS. After 6 h of hypoxia, DiD-labeled LPs (0.5 mg/mL, 200 μL) or DiD-labeled Neu-LPs (0.5 mg/mL, 200 μL) were added to the upper chamber and incubated for 3 h. The fluorescence intensities of Neu-LPs in the supernatant, intracellular, and filtered compartments were determined using a microplate reader (SpectraMax^®^ M5, Molecular Devices).

### Mouse model of MI/R

Male C57 mice were subjected to transient ligation of the left anterior descending coronary artery for 60 min followed by reperfusion. Successful acute MI/R injury was confirmed by visual inspection of the left ventricle color and changes in the electrocardiogram. One day post ischemia–reperfusion, animals were randomized into three treatment groups (*n* = 6 mice per group): intravenous injection of: (1) 200 μL PBS, (2) LPs (0.5 mg/mL, 200 μL), or (3) Neu-LPs (0.5 mg/mL, 200 μL).

### ELISA assay

To investigate the in vivo neutralizing effects of Neu-LPs, the expression levels of inflammatory factors (TNFα, IL1β, and IL6) in ischemic hearts were measured by ELISA assays according to the manufacturer’s instructions.

### Assessment of Neu-LP targeting and biodistribution

The biodistribution of Neu-LPs was assessed according to a previous article [33]. One day after reperfusion, PBS, LPs, or Neu-LPs (0.5 mg/mL, 200 μL) were intravenously injected into MI/R model mice. At predetermined intervals (3 h, 1 day, and 3 days), the mice were sacrificed. Their hearts and other organs were harvested and imaged using an in vivo imaging system (IVIS, PerkinElmer). The fluorescence intensity of DiD was analyzed using Living Image Software. After imaging, all hearts were cryosectioned (5 μm) and imaged using a confocal microscope.

### Cardiac function assessment

The left ventricular function of MI/R model mice with various treatments (PBS, LPs, or Neu-LPs) were analyzed by echocardiography (Vevo 770, Visual Sonics, Toronto, ON, Canada) 4 h post reperfusion and 4 weeks after treatment. The model mice were anesthetized with low-dose isoflurane for echocardiographic examination. Two-dimensional targeted M-mode traces were obtained at the level of papillary muscle. LVEF, FS, LVEDV, and LVESV were measured in at least three consecutive cardiac cycles.

### Histochemical and immunohistochemical assessments

At various timepoints (3, 7, 14, and 28 d) after systemic administration of PBS, LPs, or Neu-LPs, heart tissues were harvested and cut into 5-μm paraffin-embedded sections or cryosections. Fibrous heart tissue was identified by staining sections with Masson’s Trichrome reagent according to the manufacturer's instructions. Fibrosis was imaged under an inverted fluorescence microscope. Apoptotic cells were identified by staining sections with TUNEL staining kits. Cardiomyocytes were stained with mouse anti-cardiac troponin T primary antibodies. Transcriptional regulation was identified by staining sections with rabbit anti-PH3 primary antibody. Cell proliferation was identified by staining sections with rabbit anti-Ki67 primary antibody. Vasculogenesis was identified by staining sections with rabbit anti-CD31 primary antibodies. To examine macrophage polarization, mouse anti-F4/80 and rabbit anti-CD206 primary antibodies were used. Neutrophils were identified by rabbit anti-MPO primary antibodies. FITC (488 nm) or Texas-Red (594 nm) secondary antibodies were conjoined with the related primary antibodies. DAPI was utilized to visualize cell nuclei in the sections. Images were taken with a confocal microscope and quantified using ImageJ. Flow cytometry was also employed to detect the macrophage polarization. Experimental procedures are the same as previously described and PE-anti-779 F4/80 (565410), PE-Cy7-anti-CD86 (25-0862-82), APC-anti-CD206 (17-2062-82) were used to perform Flow cytometry.

### Safety evaluation

To evaluate the safety of Neu-LPs, healthy ICR mice aged 6 weeks were intravenously injected with PBS (200 μL), LPs (0.5 mg/mL, 200 μL), or Neu-LPs (0.5 mg/mL, 200 μL) every other day for one week (*n* = 6). Blood samples were collected from the mice and biochemical indexes tests were performed. ELISA were adopted to detect the concentration of IgM in MI/R mice after PBS, LPs or Neu-LPs treatment according to the manufacturer’s instruction. For histological analyses, the major organs (heart, liver, spleen, lung, and kidney) were embedded in paraffin and stained with H&E.

### Statistical analysis

All results are expressed as mean ± standard deviation (SD). Comparisons between two groups were performed with Student’s *t*-test. One-way analysis of variance (ANOVA) was employed for comparisons among more than two groups. *P*-values less than 0.05 were considered statistically significant.

## Supplementary Information

**Additional file 1: Figure S1. **Size distribution and Transmission electron microscopy images. **A** Representative size distribution of LP and Neu-LPs. **B** Representative transmission electron microscopy images of LP and Neu-LP negatively stained with uranyl acetate. Scale bars, 50 nm.** Figure S2. **The stability of Neu-LP. Long-term stability of Neu-LP in water, monitored over 7 days (n = 3). **Figure S3. **The neutralization response and binding ability. **A** Neutralization dose response of proinflammatory factors (TNFα, IL1β, IL6, and CXCL2) by Neu LPs and LPs. *P < 0.05 and ***P < 0.001 compared with the 0mg/ml group. **B** the binding capacity of Neu LPs and LPs to inflammatory cytokines (TNFα, IL1β, IL6, and CXCL2).** Figure S4.** The evaluation of mouse MI/R model. Representative images of echocardiographic analysis of Sham mice or MI/R mice.** Figure S5. **Neu-LPs distribution in heart. Representative fluorescence imaging of Neu-LPs biodistribution in heart. Scale bars, 200 μm (yellow) and 50 μm (white). **Figure S6. **Macrophage recruitment in injured hearts. The quantitation of macrophage recruitment at day 3 post MI/R injury (n = 3). ***P < 0.001 compared with the PBS group. **Figure S7. **Heart histology at day 3. Representative **A** HE stain and **B** masson trichrome stain of the infarcted heart 3 days after treatment. Scale bars, 200 μm (red) and 1 mm (black). **Figure S8. **Cardiac function assessment. Left ventricular ejection fractions (LVEF) and fractional shortening (FS) were measured by echocardiography at different time points (baseline, 1 day, 10 days and 4 weeks) (n = 6 animals per group). *P < 0.05 and **P < 0.01 compared with the PBS group. **Figure S9. **The expression level of IgM. The expression level of IgM in the MI/R model mice following Neu-LPs injection (n = 3).

## Data Availability

The data that support the findings of this study are available from the corresponding authors upon reasonable request.

## References

[1] He L, Nguyen NB, Ardehali R, Zhou B (2020). Heart regeneration by endogenous stem cells and cardiomyocyte proliferation: controversy, fallacy, and progress. Circulation.

[2] Benjamin EJ, Virani SS, Callaway CW, Chamberlain AM, Chang AR, Cheng S, Chiuve SE, Cushman M, Delling FN, Deo R, de Ferranti SD, Ferguson JF, Fornage M, Gillespie C, Isasi CR, Jiménez MC, Jordan LC, Judd SE, Lackland D, Lichtman JH, Lisabeth L, Liu S, Longenecker CT, Lutsey PL, Mackey JS, Matchar DB, Matsushita K, Mussolino ME, Nasir K, O'Flaherty M, Palaniappan LP, Pandey A, Pandey DK, Reeves MJ, Ritchey MD, Rodriguez CJ, Roth GA, Rosamond WD, Sampson UKA, Satou GM, Shah SH, Spartano NL, Tirschwell DL, Tsao CW, Voeks JH, Willey JZ, Wilkins JT, Wu JH, Alger HM, Wong SS, Muntner P (2018). Heart disease and stroke statistics-2018 update: a report from the american heart association. Circulation.

[3] Buckley LF, Abbate A (2018). Interleukin-1 blockade in cardiovascular diseases: a clinical update. Eur Heart J.

[4] Kleveland O, Kunszt G, Bratlie M, Ueland T, Broch K, Holte E, Michelsen AE, Bendz B, Amundsen BH, Espevik T, Aakhus S, Damås JK, Aukrust P, Wiseth R, Gullestad L (2016). Effect of a single dose of the interleukin-6 receptor antagonist tocilizumab on inflammation and troponin T release in patients with non-ST-elevation myocardial infarction: a double-blind, randomized, placebo-controlled phase 2 trial. Eur Heart J.

[5] Frangogiannis NG (2014). The inflammatory response in myocardial injury, repair, and remodelling. Nat Rev Cardiol.

[6] Dai Y, Song J, Li W, Yang T, Yue X, Lin X, Yang X, Luo W, Guo J, Wang X, Lai S, Andrade KC, Chang J (2019). RhoE fine-tunes inflammatory response in myocardial infarction. Circulation.

[7] Martini E, Stirparo GG, Kallikourdis M (2018). Immunotherapy for cardiovascular disease. J Leukoc Biol.

[8] Westman PC, Lipinski MJ, Luger D, Waksman R, Bonow RO, Wu E, Epstein SE (2016). Inflammation as a driver of adverse left ventricular remodeling after acute myocardial infarction. J Am Coll Cardiol.

[9] Kim ND, Luster AD (2015). The role of tissue resident cells in neutrophil recruitment. Trends Immunol.

[10] Ruparelia N, Godec J, Lee R, Chai JT, Dall'Armellina E, McAndrew D, Digby JE, Forfar JC, Prendergast BD, Kharbanda RK, Banning AP, Neubauer S, Lygate CA, Channon KM, Haining NW, Choudhury RP (2015). Acute myocardial infarction activates distinct inflammation and proliferation pathways in circulating monocytes, prior to recruitment, and identified through conserved transcriptional responses in mice and humans. Eur Heart J.

[11] Silvestre-Roig C, Braster Q, Ortega-Gomez A, Soehnlein O (2020). Neutrophils as regulators of cardiovascular inflammation. Nat Rev Cardiol.

[12] Van Tassell BW, Toldo S, Mezzaroma E, Abbate A (2013). Targeting interleukin-1 in heart disease. Circulation.

[13] Hu CM, Zhang L, Aryal S, Cheung C, Fang RH, Zhang L (2011). Erythrocyte membrane-camouflaged polymeric nanoparticles as a biomimetic delivery platform. Proc Natl Acad Sci USA.

[14] He Y, Li R, Li H, Zhang S, Dai W, Wu Q, Jiang L, Zheng Z, Shen S, Chen X, Zhu Y, Wang J, Pang Z (2019). Erythroliposomes: integrated hybrid nanovesicles composed of erythrocyte membranes and artificial lipid membranes for pore-forming toxin clearance. ACS Nano.

[15] Park JH, Dehaini D, Zhou J, Holay M, Fang RH, Zhang L (2020). Biomimetic nanoparticle technology for cardiovascular disease detection and treatment. Nanoscale Horiz.

[16] Lämmermann T (2016). In the eye of the neutrophil swarm-navigation signals that bring neutrophils together in inflamed and infected tissues. J Leukoc Biol.

[17] Parodi A, Molinaro R, Sushnitha M, Evangelopoulos M, Martinez JO, Arrighetti N, Corbo C, Tasciotti E (2017). Bio-inspired engineering of cell- and virus-like nanoparticles for drug delivery. Biomaterials.

[18] Kolaczkowska E, Kubes P (2013). Neutrophil recruitment and function in health and inflammation. Nat Rev Immunol.

[19] Margraf A, Ley K, Zarbock A (2019). Neutrophil recruitment: from model systems to tissue-specific patterns. Trends Immunol.

[20] Newby LK (2019). Inflammation as a treatment target after acute myocardial infarction. N Engl J Med.

[21] Hasler P, Giaglis S, Hahn S (2016). Neutrophil extracellular traps in health and disease. Swiss Med Wkly.

[22] Molinaro R, Corbo C, Martinez JO, Taraballi F, Evangelopoulos M, Minardi S, Yazdi IK (2016). Biomimetic proteolipid vesicles for targeting inflamed tissues. Nat Mater.

[23] Andreadou I, Cabrera-Fuentes HA, Devaux Y, Frangogiannis NG, Frantz S, Guzik T, Liehn EA, Gomes CPC, Schulz R, Hausenloy DJ (2019). Immune cells as targets for cardioprotection: new players and novel therapeutic opportunities. Cardiovasc Res.

[24] Nian M, Lee P, Khaper N, Liu P (2004). Inflammatory cytokines and postmyocardial infarction remodeling. Circ Res.

[25] Horckmans M, Ring L, Duchene J, Santovito D, Schloss MJ, Drechsler M, Weber C, Soehnlein O, Steffens S (2017). Neutrophils orchestrate post-myocardial infarction healing by polarizing macrophages towards a reparative phenotype. Eur Heart J.

[26] Nahrendorf M, Swirski FK (2013). Monocyte and macrophage heterogeneity in the heart. Circ Res.

[27] Nahrendorf M, Swirski FK, Aikawa E, Stangenberg L, Wurdinger T, Figueiredo JL, Libby P, Weissleder R, Pittet MJ (2007). The healing myocardium sequentially mobilizes two monocyte subsets with divergent and complementary functions. J Exp Med.

[28] Nahrendorf M, Pittet MJ, Swirski FK (2010). Monocytes: protagonists of infarct inflammation and repair after myocardial infarction. Circulation.

[29] Futosi K, Fodor S, Mócsai A (2013). Neutrophil cell surface receptors and their intracellular signal transduction pathways. Int Immunopharmacol.

[30] Zhang Q, Dehaini D, Zhang Y, Zhou J, Chen X, Zhang L, Fang RH, Gao W, Zhang L (2018). Neutrophil membrane-coated nanoparticles inhibit synovial inflammation and alleviate joint damage in inflammatory arthritis. Nat Nanotechnol.

[31] Hitscherich PG, Xie LH, Del Re D, Lee EJ (2019). The effects of macrophages on cardiomyocyte calcium-handling function using in vitro culture models. Physiol Rep.

[32] Liang CC, Park AY, Guan JL (2007). In vitro scratch assay: a convenient and inexpensive method for analysis of cell migration in vitro. Nat Protoc.

[33] Chen J, Song Y, Huang Z, Zhang N, Xie X, Liu X, Yang H, Wang Q, Li M, Li Q, Gong H, Qian J, Pang Z, Ge J (2019). Modification with CREKA improves cell retention in a rat model of myocardial ischemia reperfusion. Stem Cells.

