# Supplementary material for: Targeted neutrophil-mimetic liposomes promote cardiac repair by adsorbing proinflammatory cytokines and regulating the immune microenvironment
Source: J Nanobiotechnology. 2022 May 7;20:218. doi: 10.1186/s12951-022-01433-6 (PMC9077972; doi:10.1186/s12951-022-01433-6)
Supplement: Supplementary file 1 — Additional file 1: Figure S1. Size distribution and Transmission electron microscopy images. A Representative size distribution of LP and Neu-LPs. B Representative transmission electron microscopy images of LP and Neu-LP negatively stained with uranyl acetate. Scale bars, 50 nm. Figure S2. The stability of Neu-LP. Long-term stability of Neu-LP in water, monitored over 7 days (n = 3). Figure S3. The neutralization response and binding ability. A Neutralization dose response of proinflammatory factors (TNFα, IL1β, IL6, and CXCL2) by Neu LPs and LPs. *P < 0.05 and ***P < 0.001 compared with the 0mg/ml group. B the binding capacity of Neu LPs and LPs to inflammatory cytokines (TNFα, IL1β, IL6, and CXCL2). Figure S4. The evaluation of mouse MI/R model. Representative images of echocardiographic analysis of Sham mice or MI/R mice. Figure S5. Neu-LPs distribution in heart. Representative fluorescence imaging of Neu-LPs biodistribution in heart. Scale bars, 200 μm (yellow) and 50 μm (white). Figure S6. Macrophage recruitment in injured hearts. The quantitation of macrophage recruitment at day 3 post MI/R injury (n = 3). ***P < 0.001 compared with the PBS group. Figure S7. Heart histology at day 3. Representative A HE stain and B masson trichrome stain of the infarcted heart 3 days after treatment. Scale bars, 200 μm (red) and 1 mm (black). Figure S8. Cardiac function assessment. Left ventricular ejection fractions (LVEF) and fractional shortening (FS) were measured by echocardiography at different time points (baseline, 1 day, 10 days and 4 weeks) (n = 6 animals per group). *P < 0.05 and **P < 0.01 compared with the PBS group. Figure S9. The expression level of IgM. The expression level of IgM in the MI/R model mice following Neu-LPs injection (n = 3). [file 12951_2022_1433_MOESM1_ESM.docx]

**Additional file 1**

**A**

LP Neu-LP


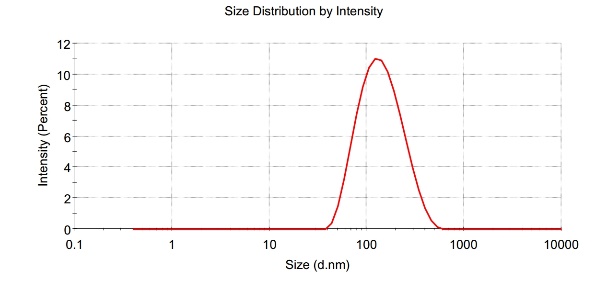

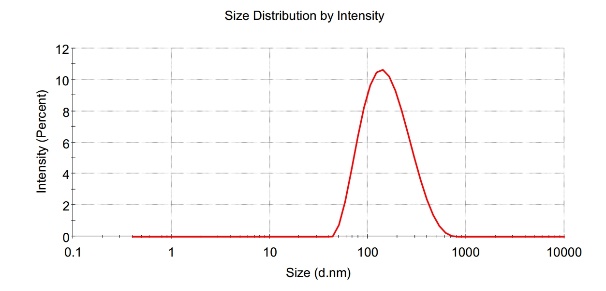


**B**

LP Neu-LP


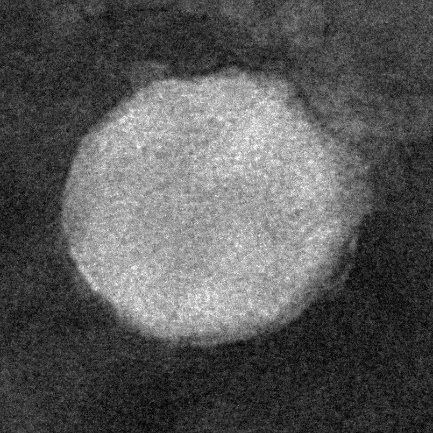
 **
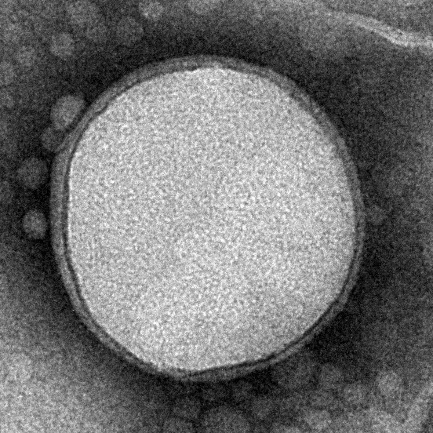
**

**Figure S1. Size distribution and Transmission electron microscopy images.**

(A) Representative size distribution of LP and Neu-LPs. (B) Representative transmission electron microscopy images of LP and Neu-LP negatively stained with uranyl acetate. Scale bars, 50 nm.

**Figure S2. The stability of Neu-LP .**

Long-term stability of Neu-LP in water, monitored over 7 days (n = 3).

**
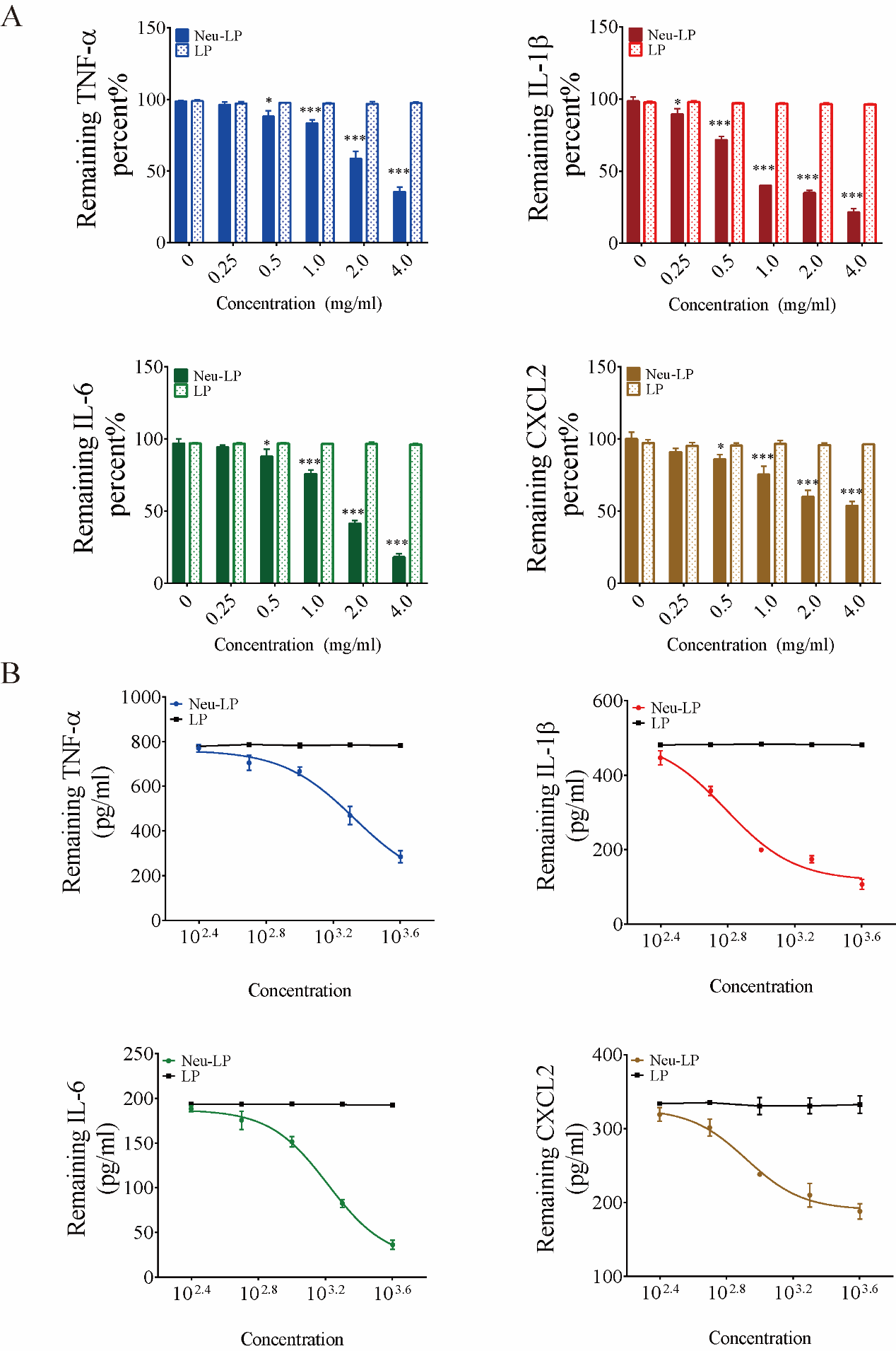
**

**Figure S3. The neutralization response and binding ability.**

1. Neutralization dose response of proinflammatory factors (TNFα, IL1β, IL6, and CXCL2) by Neu LPs and LPs. *P < 0.05 and ***P < 0.001 compared with the 0mg/ml group. (B) the binding capacity of Neu LPs and LPs to inflammatory cytokines (TNFα, IL1β, IL6, and CXCL2).

**
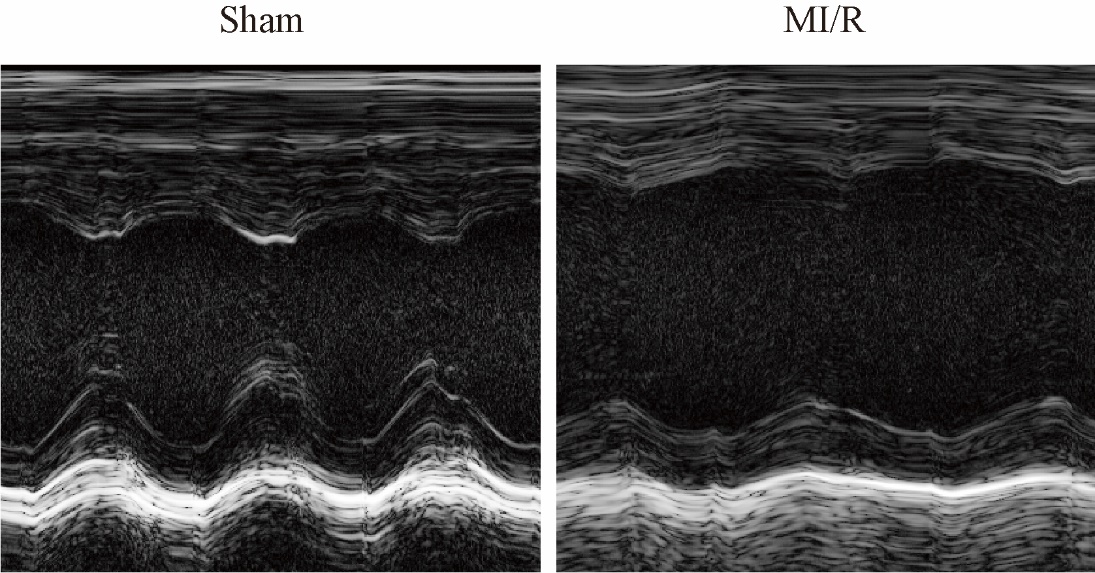
**

**Figure S4 . The evaluation of mouse MI/R model .**

Representative images of echocardiographic analysis of Sham mice or MI/R mice .


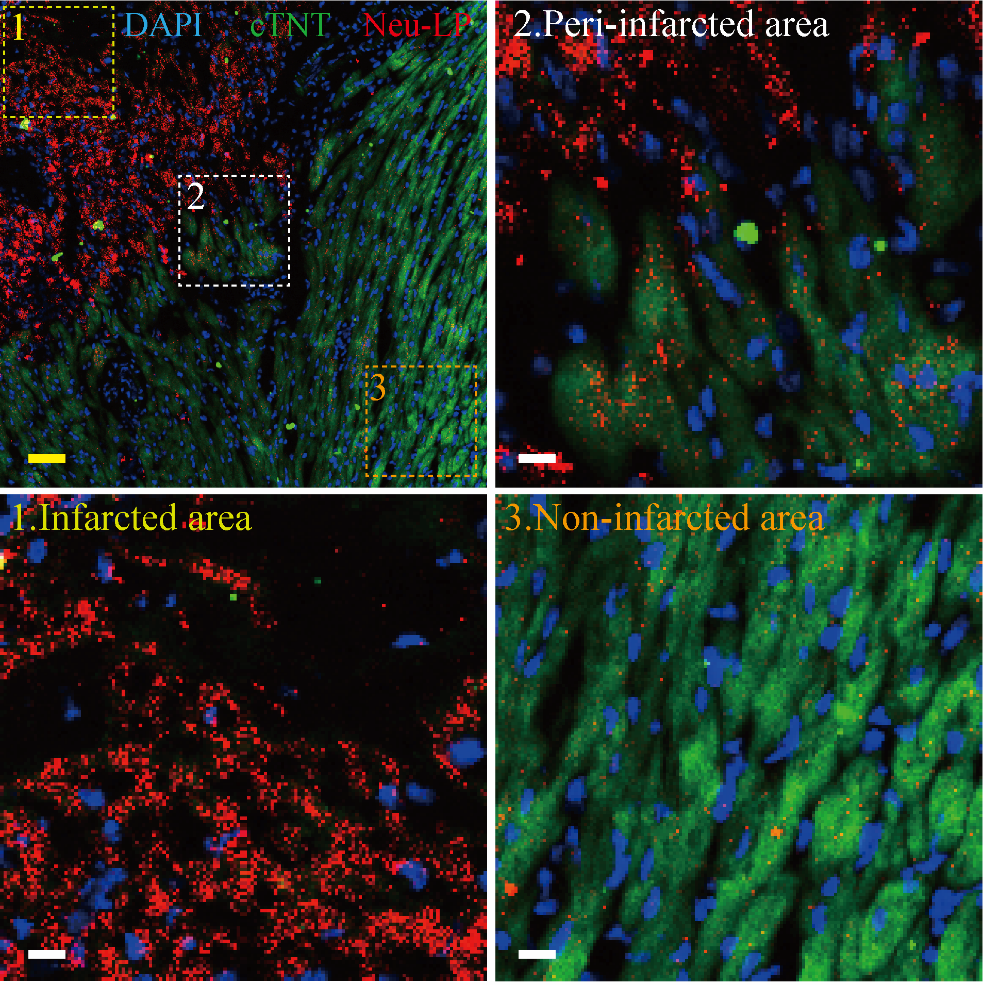


**Figure S5. Neu-LPs distribution in heart.**

Representative fluorescence imaging of Neu-LPs biodistribution in heart. Scale bars, 200 μm (yellow) and 50 μm (white).

**Figure S6. Macrophage recruitment in injured hearts .**

The quantitation of macrophage recruitment at day 3 post MI/R injury (n = 3). ***P < 0.001 compared with the PBS group.

**
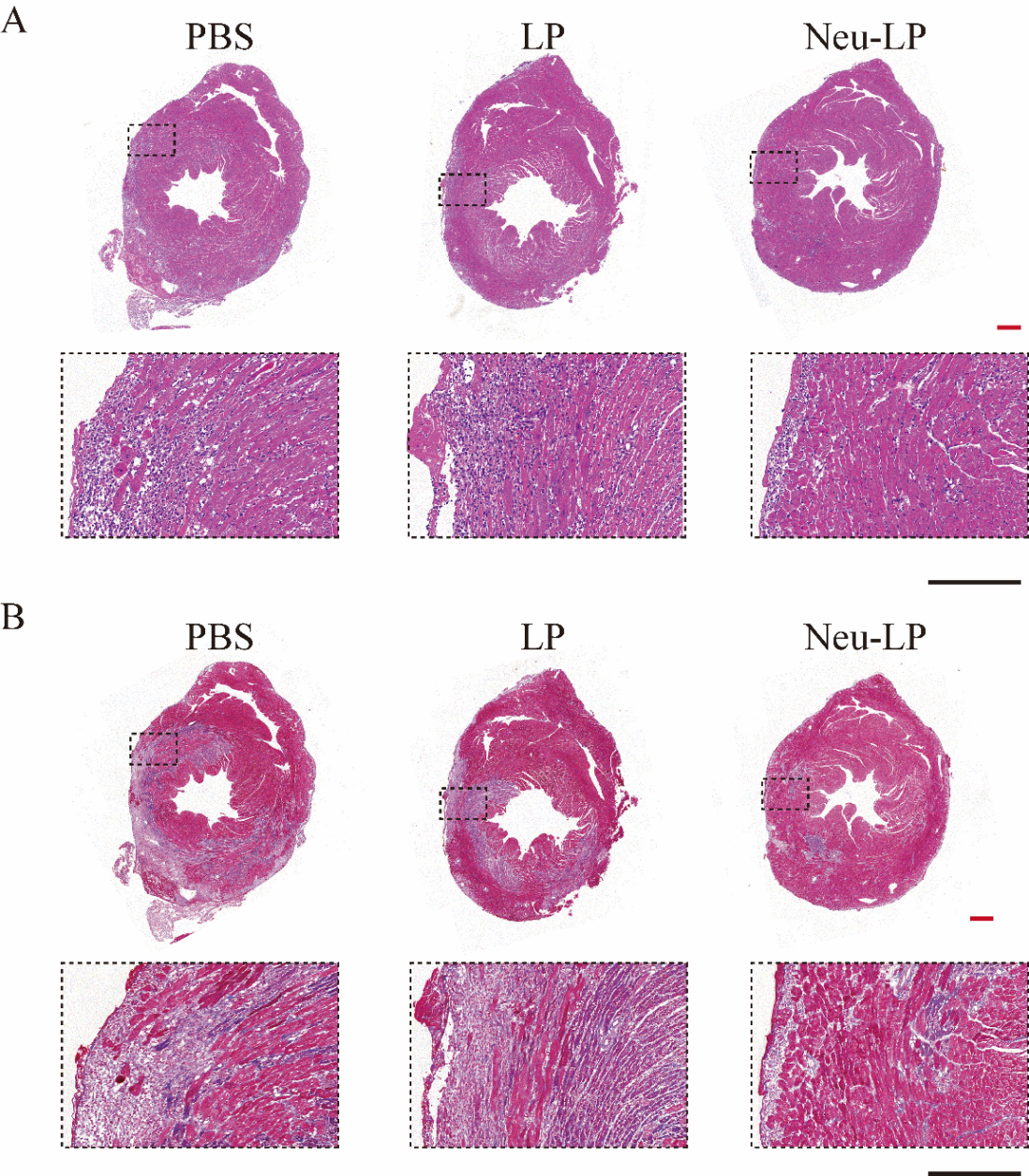
**

**Figure S7. Heart histology at day 3.**

Representative (A) HE stain and (B) masson trichrome stain of the infarcted heart 3 days after treatment. Scale bars, 200 μm (red) and 1 mm (black).


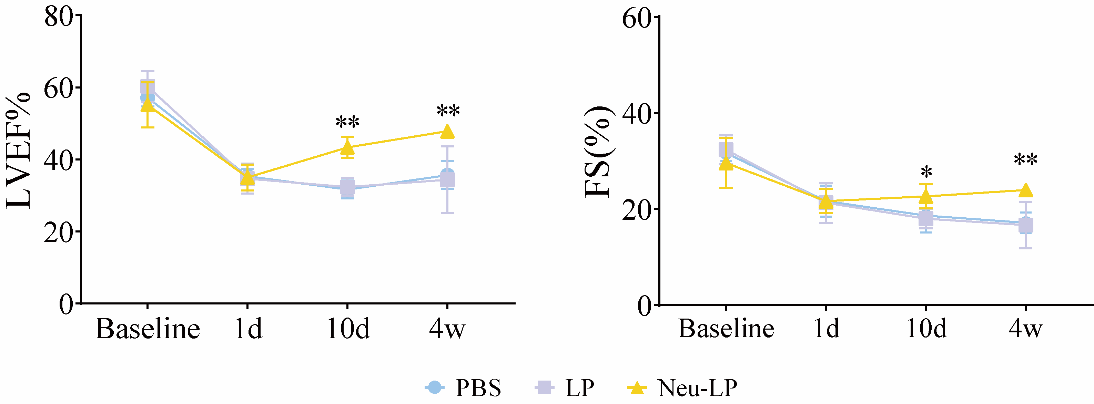


**Figure S8. Cardiac function assessment.**

Left ventricular ejection fractions (LVEF) and fractional shortening (FS) were measured by echocardiography at different time points (baseline, 1 day, 10 days and 4 weeks) (n = 6 animals per group). *P < 0.05 and **P < 0.01 compared with the PBS group.

**Figure S9. The expression level of IgM.**

The expression level of IgM in the MI/R model mice following Neu-LPs injection (n = 3).
